# Hydrogen production from water electrolysis: role of catalysts

**DOI:** 10.1186/s40580-021-00254-x

**Published:** 2021-02-11

**Authors:** Shan Wang, Aolin Lu, Chuan-Jian Zhong

**Affiliations:** Department of Chemistry, State University of New York at Binghamton, Binghamton, NY 13902 USA

**Keywords:** Electrocatalysts, Water splitting electrolysis, Hydrogen production, Energy storage and conversion, Oxygen evolution reaction, Hydrogen evolution reaction

## Abstract

As a promising substitute for fossil fuels, hydrogen has emerged as a clean and renewable energy. A key challenge is the efficient production of hydrogen to meet the commercial-scale demand of hydrogen. Water splitting electrolysis is a promising pathway to achieve the efficient hydrogen production in terms of energy conversion and storage in which catalysis or electrocatalysis plays a critical role. The development of active, stable, and low-cost catalysts or electrocatalysts is an essential prerequisite for achieving the desired electrocatalytic hydrogen production from water splitting for practical use, which constitutes the central focus of this review. It will start with an introduction of the water splitting performance evaluation of various electrocatalysts in terms of activity, stability, and efficiency. This will be followed by outlining current knowledge on the two half-cell reactions, hydrogen evolution reaction (HER) and oxygen evolution reaction (OER), in terms of reaction mechanisms in alkaline and acidic media. Recent advances in the design and preparation of nanostructured noble-metal and non-noble metal-based electrocatalysts will be discussed. New strategies and insights in exploring the synergistic structure, morphology, composition, and active sites of the nanostructured electrocatalysts for increasing the electrocatalytic activity and stability in HER and OER will be highlighted. Finally, future challenges and perspectives in the design of active and robust electrocatalysts for HER and OER towards efficient production of hydrogen from water splitting electrolysis will also be outlined.

## Introduction

**Fig. 1:**
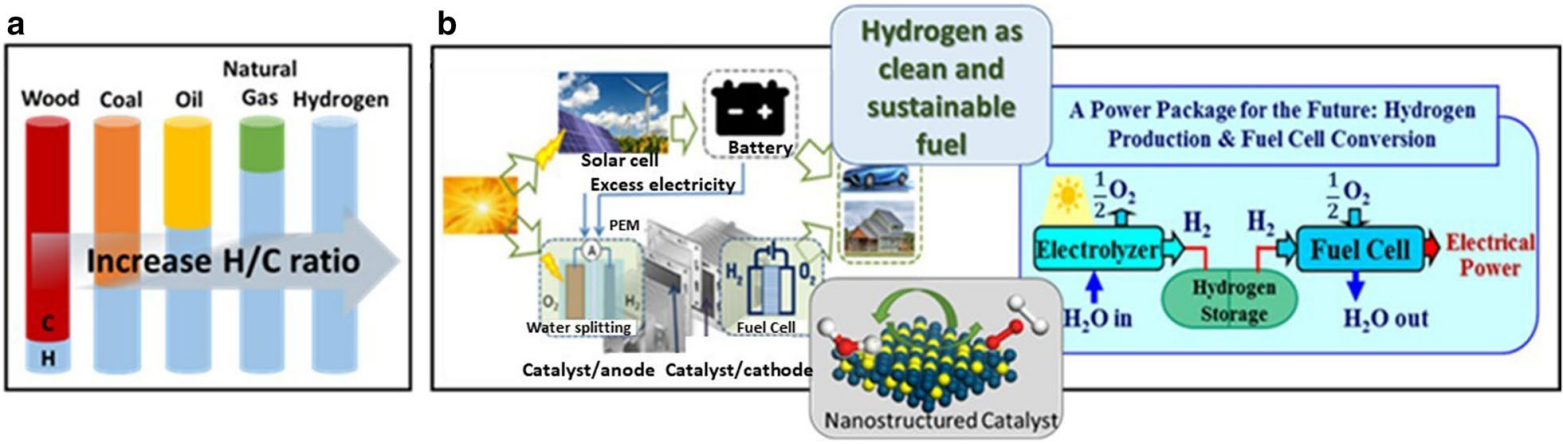
**a** An illustration of the evolution of fuels in terms of hydrogen to carbon ratio. **b** Illustrations of a dual cell functioning as an electrolysis water splitting cell for hydrogen production from solar energy and a fuel cell for the conversion of hydrogen to electricity, highlighting the sustainable power package of the future and the role of catalysis

Energy and environment are two key issues in modern society which are necessities for the economic and social sustainable development of the world [1, 2]. In 2018, there is 79.5% energy economy that relies on conventional energy sources such as coal, petroleum oil, and natural gas, which are not renewable and environmentally benign [3]. To deal with this problem, there has been a global drive seeking renewable and clean alternatives to fossil fuels. Nature offers various renewable sources such as solar energy, wind energy, tidal energy, biomass energy, etc. However, such energy sources suffer from intermittent availability due to regional or seasonal factors [4]. As a result, an efficient energy conversion and storage system is required in conjunction with the exploration of renewable energy sources for large scale utilization [1]. This need constitutes a major driving force for numerous innovations in energy conversion and storage systems. Indeed, systems such as hydrogen production from water splitting by electrolysis, fuel cells for converting hydrogen to electricity, and lithium-ion or metal-air batteries for energy storage have drawn a great deal of attention in recent decades [5]. For the battery-based energy storage, it is increasingly difficult to store excess electricity from a large-scale production facility, which is very expensive and needs a large facility area. With the large-scale solar or wind produced excess electricity, an alternative pathway for energy storage is needed. Hydrogen production by electricity-driven water splitting has become a promising strategy to convert the large excess amount of electrical energy from the renewable energy resources in the form of a clean fuel—hydrogen (H_2_). As a clean and sustainable energy carrier, hydrogen has the highest gravimetric energy density. When it is used as the fuel in a fuel cell, it features not only high efficiency in energy conversion, but also produces zero pollution as it emits only water as a byproduct. Therefore, the development of water splitting cells for hydrogen production from renewable sources and fuel cells for effective conversion of hydrogen to electricity has become a global drive towards a sustainable power package of the future (Fig. 1). This development is leading its way to address many of the challenging issues facing energy and environmental sustainability. Significant progress has been made in the fronts of electrolysis water-splitting cells [1, 2, 5–11] and fuel cells [12–18], offering hopes for a sustainable transition to carbon–neutral operations.

Among many aspects of the progress in the development of the sustainable power package of the future, catalysis, or electrocatalysis, has played a major role in overcoming the kinetic energy barriers for electrochemical reactions of water, oxygen, and hydrogen in water-splitting cells and fuel cells (Fig. 1). It is the role of catalysis in electrolysis water-splitting cells that is the focal point of this review. The readers are referred to several recent reviews for the role of catalysis in fuel cells [15, 19–26].

Generally, the overall reaction of water electrolysis can be divided into two half-cell reactions: hydrogen evolution reaction (HER) and oxygen evolution reaction (OER). HER is the reaction where water is reduced at the cathode to produce H_2_, and OER is the reaction where water is oxidized at the anode to produce O_2_. One of the critical barriers that keep water splitting from being of practical use is the sluggish reaction kinetics of OER and HER due to high overpotentials[5], a measure of the kinetic energy barriers. Therefore, catalysis plays a major role in both OER and HER. Highly effective catalysts are required to minimize the overpotentials for OER and HER towards efficient H_2_ and O_2_ production.

The design of catalysts or electrocatalysts depends on the operating conditions of the water electrolysis cell. Currently, there are three main types of electrolysis technologies: (1) proton exchange membrane (PEM) electrolysis (2) alkaline electrolysis (3) high-temperature solid oxide water electrolysis. The solid oxide water electrolysis requires high energy consumption because of the high temperature. For the PEM based electrolysis cell, the water splitting is performed under acidic condition and using PEM. This condition has some advantages over other conditions such as lower gas permeability and high proton conductivity. It features high energy efficiency and fast hydrogen production rate [8]. However, the requirement of acidic media limits the OER electrocatalysts to noble metal and noble metal oxide catalysts, which are the state-of-the-art OER electrocatalysts in the acidic media. This requirement leads to a high cost for the cell [27]. For the alkaline electrolysis cell, water splitting is performed under alkaline condition. In comparison with cells using acidic media, water splitting in alkaline media broadens the selection of the electrocatalysts to non-noble metals or metal oxides. However, the activity of HER in alkaline media is usually 2–3 orders of magnitude lower than the activity of HER in acidic media [28]. Therefore, the design of optimal electrocatalysts suitable for the different media with low-cost, high catalytic activity, and good durability for electrolytic water splitting is very challenging.

Because of the surge of recent interest in hydrogen production from water electrolysis, there have been many excellent reviews describing the progress of research in HER and OER [4, 8, 29]. Many of the reviews focused on a comprehensive overview in terms of the half-cell reaction mechanisms in alkaline media, and the progress in the preparation of noble metal-free electrocatalysts which exhibit excellent catalytic activity and durability for HER and OER. There is a relatively limited number of reviews discussing insights into mechanistic details in terms of catalysts’ structure, morphology, composition, active site, and their correlation with activity and stability for HER and OER in both acidic and alkaline media, which constitutes the focus of the present review. This focus stems from the increasing need of active, stable, and low-cost electrocatalysts for the efficient production of hydrogen from electrocatalytic water splitting. There are several challenging areas for the development of active, stable, and low-cost electrocatalysts. First, while most of the efficient OER catalysts such as Ir and Ru based electrocatalysts exhibit a high dissolution resistance in acidic condition, most of the non-noble metal-based electrocatalysts cannot survive under such condition. Thus, the challenge is to develop stable and robust non-noble metal OER electrocatalysts with high activity and long-term stability performance in acidic media. Second, while non-noble-metal-based electrocatalysts such as carbides, phosphides, and chalcogenides have drawn great attention due to their high performance for OER in alkaline media, the catalysts undergo composition and structural transformation during OER condition. Thus, the identification of the real active sites remains elusive, and it is challenging to develop techniques to detect the real active sites to guide the design and preparation of optimal catalysts. Third, the knowledge on the catalytic mechanisms of many electrocatalysts, especially transition metal-based catalysts for HER in alkaline condition, is rather limited in comparison with HER in acidic condition. Thus, an important challenge is to determine the factors that govern the catalytic mechanism of HER in alkaline media.

In this review, we start with an introduction of the performance index used to evaluate the electrocatalysts’ activity, stability, and efficiency. It is followed by discussions of HER reaction pathways and mechanisms in acidic and alkaline media. We will discuss the recent advances in developing strategies for performance improvement of HER electrocatalysts derived from noble metal and non-noble-metal-based metal carbides, metal phosphides, and metal chalcogenides. In the subsequent section, we discuss recent insights into the mechanistic details of the catalysts and possible reaction pathways in acidic media and alkaline media. This discussion is followed by highlighting some recent development in increasing the intrinsic activity of active sites on nanostructured noble-metal-based and non-noble-metal-based catalysts for OER. Some of the recent insights into the lattice oxygen mediated mechanism (LOM) will also be highlighted. Finally, future challenges and perspectives in the design of nanostructured electrocatalysts for HER and OER towards efficient production of hydrogen from water splitting by electrolysis will also be outlined.

## Performance evaluation index for electrocatalysts

**Fig. 2:**
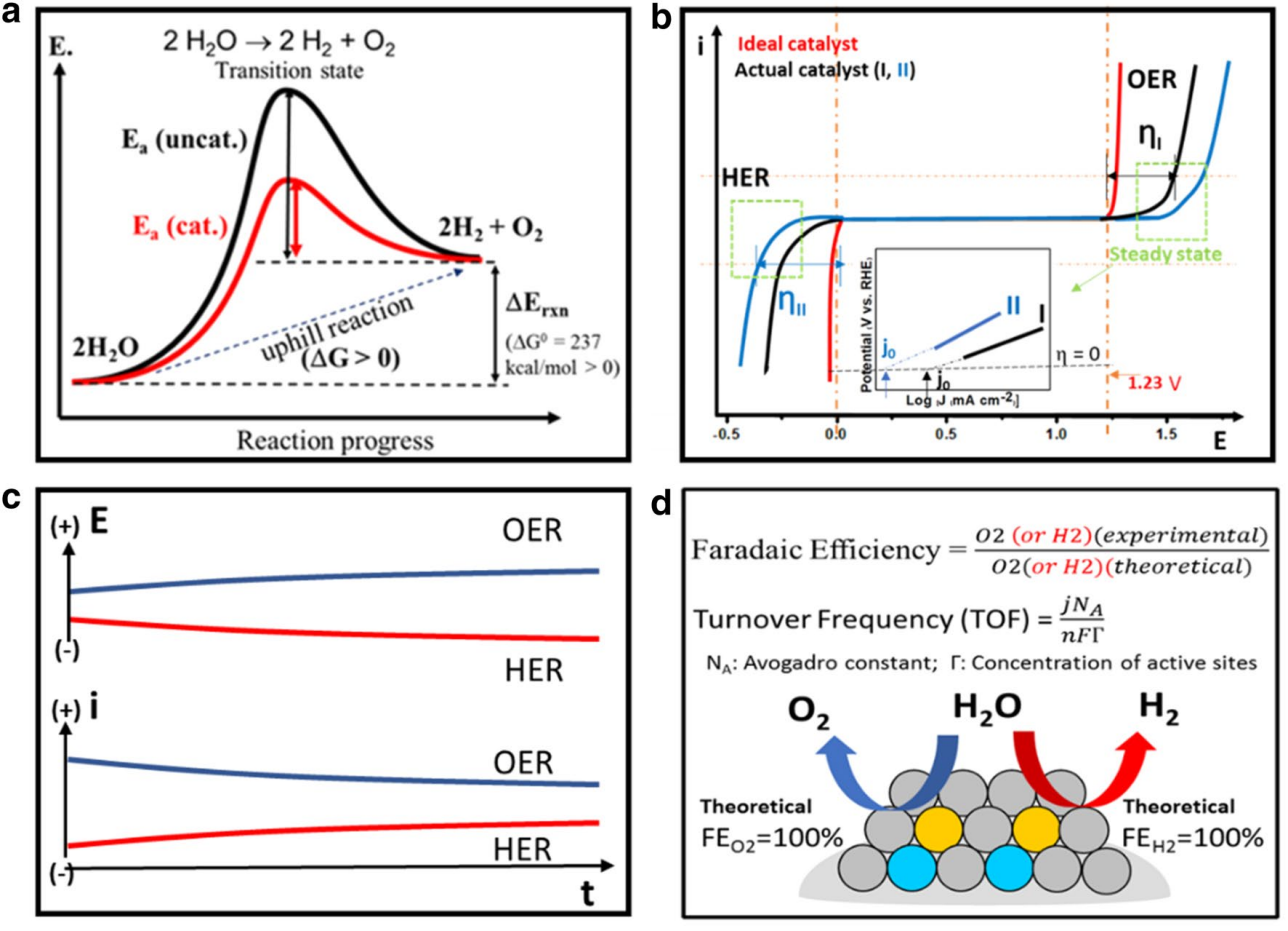
**a** Schematic illustration of the catalyst’s role in lowering the activation energy barrier; **b**–**d** schematic illustrations of the performance evaluation parameters of electrocatalyst, including, **b** activity in terms of overpotential, Tafel slope, and exchange current density, **c** stability in terms of current- and potential-time curves, and **d** efficiency in terms of faradaic efficiency and turnover frequency

Electrolytic water splitting is not only an uphill reaction, as reflected by the positive value of ΔG (Gibbs free energy), but also has to overcome a significant kinetic barrier. Catalysts play a crucial role in lowering the kinetic barrier (Fig. 2a). The evaluation of the performance of a catalyst for the electrocatalytic water splitting is based on several key parameters for activity, stability, and efficiency (Fig. 2). The activity is characterized by overpotential, Tafel slope, and exchange current density, which can be extracted from the polarization curves (Fig. 2b). The stability is characterized by the changes of the overpotential or current over time (Fig. 2c). The efficiency is characterized by the faradaic efficiency and turnover frequency in terms of experimental results vs. theoretical predictions (Fig. 2d).

### Activity in terms of overpotential, Tafel slope, and exchange current density

For electrochemical water splitting reaction, the thermodynamic potential is 1.23 V at 25 °C and 1 atm. However, due to the kinetic barrier for the reaction, water electrolysis requires a higher potential than thermodynamic potential (1.23 V) to overcome the kinetic barrier. The excess potential is also known as overpotential (η) which mainly comes from the intrinsic activation barriers present on both anode and cathode. Overpotential is a very important descriptor to evaluate the activity of the electrocatalysts. Usually, the overpotential value corresponding to the current density of 10 mA cm^−2^ is used to compare the activities among different catalysts. This current density corresponds to a 12.3% solar-to-hydrogen efficiency.

The Tafel slope and exchange current are two other parameters to assess the activity from the overpotential vs. kinetic current relationship, which is expressed by the equation: η = a + b log j, where η is the overpotential, and j is the current density. In the Tafel plot, the linear correlation yields two important kinetic parameters. One is the Tafel slope b, and the other is the exchange current density j_0_ which can be obtained by extracting the current at zero overpotential. The Tafel slope b is related to the catalytic reaction mechanism in terms of electron-transfer kinetics. For example, a smaller Tafel slope means that there is a significant current density increment as a function of the overpotential change, or in other words, faster electrocatalytic reaction kinetics. The exchange current density describes the intrinsic charge transfer under equilibrium conditions. A higher exchange current density means a greater charge transfer rate and a lower reaction barrier. A lower Tafel slope and a higher exchange current density are expected for a better electrocatalyst.

### Catalyst stability in terms of current- and potential-time curves

Stability is an important parameter to evaluate whether the catalyst has the potential for use in water splitting cells in practical applications. There are two typical methods for characterizing the stability of electrocatalysts. One method is chronoamperometry (I–t curve) or chronopotentiometry (E–t curve) which measures the current variation with time under a fixed potential or measure the potential change with time at a fixed current. For this measurement, the longer the tested current or potential remains constant, the better the stability of the catalyst. For the comparison with the different research groups, people usually set a current density larger than 10 mA cm^−2^ for at least 10 h test. Another method is cyclic voltammetry (CV) which measures the current by cycling the potential, usually requiring more than 5000 cycles of run at a scan rate (e.g. 50 mV s^−1^). Linear sweep voltammetry (LSV) is typically applied to examine the overpotential shift before and after CV cycling at a specific current density. The smaller the change of overpotential is, the better the electrocatalyst’s stability.

### Efficiency in terms of faradaic efficiency and turnover frequency

Faradaic efficiency is a quantitative parameter used to describe the efficiency of electrons in the external circuit that is transferred to the electrode surface for the electrochemical reaction. The definition of faradaic efficiency is the ratio of the experimentally detected quantity of H_2_ or O_2_ to the theoretically calculated quantity of H_2_ or O_2_. The theoretical values can be calculated from the integration of the chronoamperometric or chronopotentiometric analysis. The experimental values are measured by analyzing the gas production using the water–gas displacement method or gas chromatography method.

Turnover frequency (TOF) is a useful parameter to describe the reaction rate in terms of the catalytic sites which is the intrinsic catalytic activity of the catalyst. In general, TOF describes how many reactants can be converted to the desired product per catalytic site per unit time. However, it is usually difficult to calculate the precise TOF value for most heterogeneous electrocatalysts since the precise number of active sites per electrode area is often an estimate. Despite being relatively imprecise, TOF still is a useful way to compare the catalytic activities among different catalysts, especially within a similar system or under a similar condition.

The choice of the techniques for the analysis of activity, stability, and efficiency of the catalysts depends on the specific focus of the research and development. In addition to synthesis and preparation of the electrocatalysts, the current studies of the activity, stability, and efficiency can be grouped in three areas in terms of the specific focus: performance evaluation, structural characterization, and mechanistic determination. While the analysis of the current- or potential-time curves provides information for assessing the catalyst’s durability performance, which is very important for practical applications, the determination of the overpotential, Tafel slope, exchange current density, faradaic efficiency, turnover frequency, and provides the basic parameters for assessing the electrocatalytic mechanism. Importantly, coupling of these electrochemical techniques to spectroscopic and microscopic techniques (ex-situ or in-situ) enables the structural characterization, which is crucial for gaining insights into the design of the active and stable catalysts. Examples in terms of the rationale of applying the different techniques for the specific research and development focus will be highlighted in the following subsections.

## Electrocatalysts for hydrogen evolution reaction (HER)

### Reaction steps in HER

HER is the key half-reaction to produce hydrogen at the cathode in water electrolysis which involves a two-electron transfer process. The mechanism of this HER is highly dependent on the environmental condition. For the HER reaction in acidic media, there are three possible reaction steps.

1a $$ {\text{H}}^{ + } + {\text{e}}^{ - } = {\text{H}}_{{\text{ad }}} , $$

1b $$ {\text{H}}^{ + } + {\text{e}}^{ - } + {\text{H}}_{{{\text{ad}}}} = {\text{H}}_{2} , $$

1c $$ 2{\text{H}}_{{{\text{ad}}}} = {\text{H}}_{2} . $$

The first step is Volmer step (1a) to produce adsorbed hydrogen_._ Then, the hydrogen evolution reaction can proceed by Heyrovsky step (1b) or the Tafel step (1c) or both to produce H_2_ [30]. For the HER reaction in alkaline media. There are two possible reaction steps, i.e., Volmer step (2a) and Heyrovsky step (2b) [31], as shown in the following equations, respectively:

2a $$ {\text{H}}_{2} {\text{O}} + {\text{e}}^{ - } = {\text{OH}}^{ - } + {\text{H}}_{{{\text{ad}}}} , $$

2b $$ {\text{H}}_{2} {\text{O}} + {\text{e}}^{ - } + {\text{H}}_{{{\text{ad}}}} = {\text{OH}}^{ - } + {\text{H}}_{2} . $$

It is vital to trade-off H_ad_, hydroxy adsorption (OH_ad_), and water dissociation for HER activity in alkaline media.

**Fig. 3:**
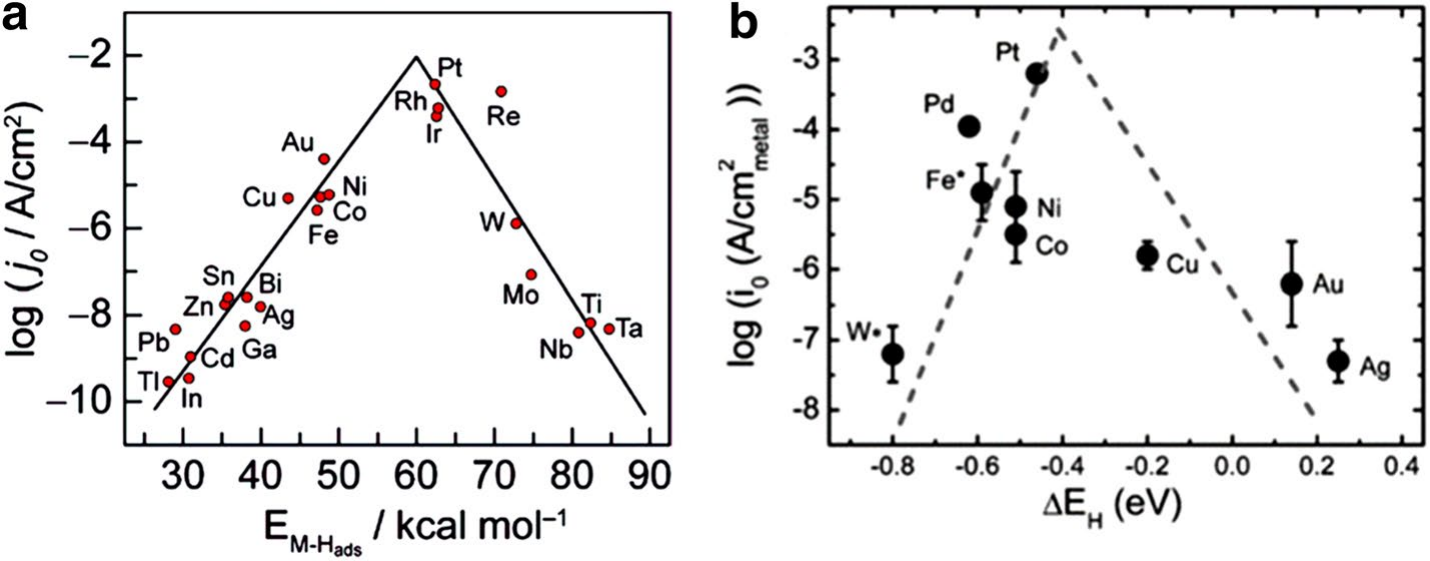
Volcano plots: **a** exchange current density vs. the M–H bond energy for each metal surface (for acidic media) (Figure reprinted with permission from Refs. [32, 33]); **b** exchange current density on monometallic surfaces vs. the calculated HBE (for alkaline media) (Figure reprinted with permission from Ref. [34])

Theoretical simulations have revealed that HER activity was related to hydrogen adsorption (H_ad_). The free energy of hydrogen adsorption (ΔG_H_) is widely accepted to be a descriptor for a hydrogen evolution material. A moderate value of hydrogen binding energy will benefit HER process. As shown in Fig. 3a [32, 33], and Fig. 3b [34], the volcano curve provides a quick comparison of the activities of different metals in acidic media and alkaline media, respectively. Pt appears to be the best catalyst for HER in both media which have the optimal hydrogen adsorption energy showing the highest exchange current density. The activity of HER in alkaline media is usually lower than that in acidic media [28]. This largely stems from the fact that the reaction is hindered by the sluggish water dissociation step, which leads to a reduction of the reaction rate by 2–3 orders of magnitude. However, alkaline electrolysis is more preferable in industrial plants. The rational design of electrocatalysts with high alkaline HER performance requires the catalysts to have the characteristics of binding hydrogen species and dissociating water.

### HER electrocatalysts

Table 1 lists some of the recent examples of studies in developing effective catalysts for HER. These catalysts are compared in terms of the electrocatalytic performance and kinetic parameters under different reaction conditions. There are two main types of HER electrocatalyst: noble-metal based electrocatalysts and non-noble metal based electrocatalysts. For the noble-metal based electrocatalyst, especially Pt-based catalysts, several strategies are being developed to increase HER performance and lower the electrocatalyst price. For example, alloying Pt with other low-cost transition metal which could improve Pt utilization and the synergistic effect of alloy could modify the electronic environments to improve the activity. Also, coupling Pt with other water dissociation promoters is an important strategy to improve the alkaline HER activities which is very meaningful for industry practical use. For non-noble metal based HER electrocatalysts, a great deal of attention has been drawn to their development largely based on the considerations of the low-cost and earth-abundant characteristics. In the following sections, we will start with a discussion of selected examples of noble metal catalysts. We will then focus on several categories of non-noble metal based electrocatalysts such as transition metal carbides, transition metal phosphides, and transition metal chalcogenides which have gone through great development in the field of electrocatalytic HER.

**Table 1:** Summary of the HER performance of the reported electrocatalysts

Catalysts	Electrolyte	η (mV)	*I* (mA cm^−2^)	Tafel slope (mV dec^−1^)	Stability	Refs.
PtNi–Ni NA/CC	0.1 M KOH	38	10	42	90 h	[35]
PtNi–O/C	1 M KOH	39.8	10	78.8	10 h	[36]
PtNi(N) NW	1 M KOH	13	10	29	10 h	[37]
Mo_2_C-R	1 M KOH	200	30	45		[38]
	0.5 M H_2_SO_4_	200	32	58	2000 cycle	
Mo_2_C-GNR	0.5 M H_2_SO_4_	167	10	63	3000 cycles	[39]
	1 M NaOH	217	10	64	3000 cycles	
	1 M PBS	266	10	74	3000 cycles	
Ni_2_P/Ti	0.5 M H_2_SO_4_	130	20	46	500 cycles	[40]
NiCo_2_P_x_	1 M KOH	58	10	34.3	5000 cycles	[41]
	1 M PBS	63	10	63.3	5000 cycles	
	0.5 M H_2_SO_4_	104	10	59.6	5000 cycles	
Defect-rich MoS_2_	0.5 M H_2_SO_4_	200	13	50	10,000 s	[42]
CoS_2_ NW	0.5 M H_2_SO_4_	145	10	51.6	3 h	[43]
CoS_2_ MW	0.5 M H_2_SO_4_	158	10	58	41 h	
Oxygenated MoS_2_	0.5 M H_2_SO_4_	120	Onset	55	3000 cycles	[44]

#### Noble-metal based electrocatalysts

Noble metals, such as Pt group metals (PGMs, including Pt, Pd, Ru, Ir, and Rh) show outstanding catalytic performance for HER. Pt ranks at the top of the volcano curve in Fig. 3. However, the commercial application of these noble-metal based catalysts is hindered by their scarce storage and high cost. To overcome this challenge, a rational design of catalysts with low metal loading and high metal utilization is necessary.

Alloying Pt with transition metal can significantly improve the Pt utilization and the synergistic effect of alloy could modify the electronic environments which significantly promote the HER electroactivity. Sun et al. reported in situ growth of ultrafine PtNi nanoparticle-decorated Ni nanosheet array on carbon cloth (PtNi-Ni NA/CC) with ultralow loading Pt content (7.7%) which show better HER activity with low overpotential 38 mV in 0.1 M KOH at the current density of 10 mA cm^−2^ than the benchmark Pt/C (20%). Remarkably, it also shows long-term durability with a 90 h catalytic activity test. The superior HER performance of PtNi–Ni NA/CC could be rationally attributed to the downshift of the d-band center of Pt, which weakens the adsorption energy of oxygenated species (OH*) on the surface Pt atom [35].

As a benchmark for Pt electrocatalyst, the activity of HER in alkaline media is usually lower than the activity of HER in acidic media [3]. The reason is the water dissociation on the Pt surface is inefficient which results in poor HER activity. To this end, coupling Pt with water dissociation promoters is the commonly used strategy to boost the alkaline HER activities [37].

**Fig. 4:**
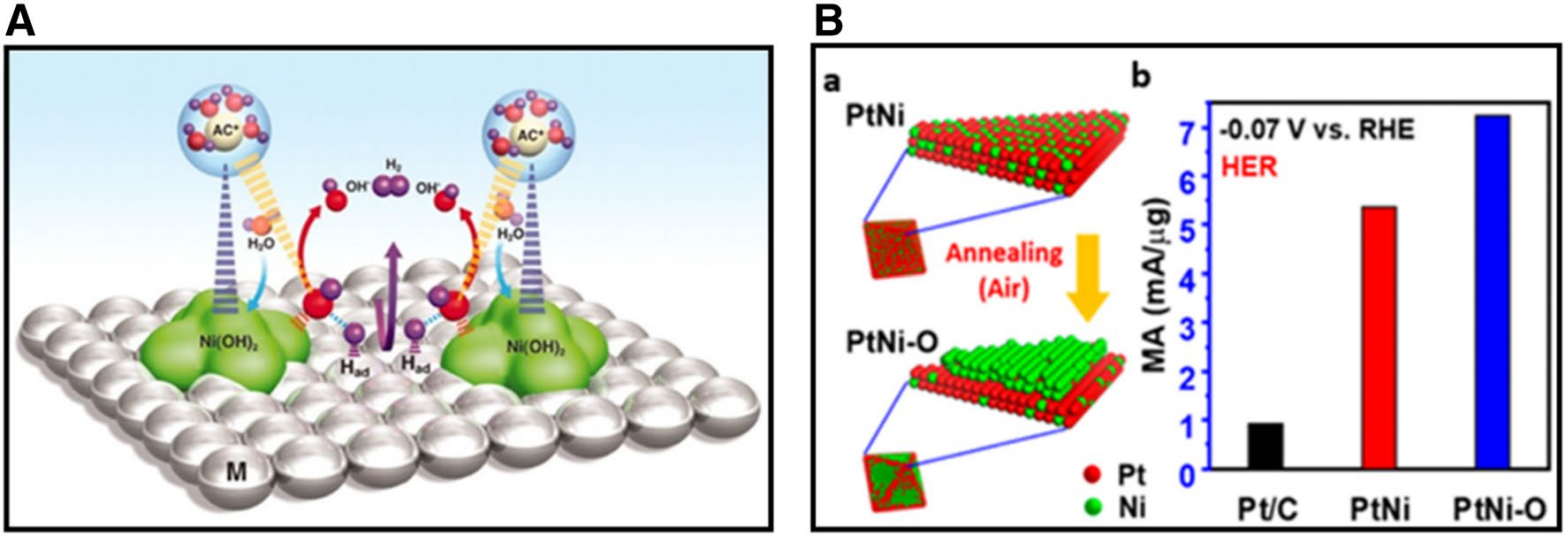
**A** Schematic illustration of the mechanism of HER on nanometer-scale Ni(OH)_2_ clusters on Pt electrode surface (Figure reprinted with permission from Ref. [48]). **B** (a) Schematic illustration of the transformation of PtNi/C to PtNi–O/C via annealing in air, and (b) comparison of HER mass activities at − 0.07 V vs RHE (Figure reprinted with permission from Ref. [36])

Among different Pt-based electrocatalysts for HER, [35, 45–47] the ability to control the metal composition on the surface is critical for enhancing the electrocatalytic activity. Recently, Markovic et al. [48] demonstrated a controlled preparation of nanometer-scale Ni(OH)_2_ clusters on Pt electrode surface, showing a factor of 8 in the enhancement of HER activity compared with state-of-the-art Pt. Figure 4A shows the proposed HER mechanism. The edges of Ni(OH)_2_ cluster promote water dissociation to form M–H_ad_ intermediates on Pt surface. The adsorbed hydrogen intermediates combine to produce H_2_. Inspired by the synergy between Ni(OH)_2_ and Pt(111), Huang et al. demonstrated the formation of surface-engineered PtNi–O nanoparticles with an enriched NiO/PtNi interface. This interface structure transforms to Ni(OH)_2_ in alkaline media, creating Ni(OH)_2_/Pt(111)-like interface on the surface. The catalyst showed a low HER overpotential of 39.8 mV at 10 mA cm^−2^ with only 5.1 µg_pt_ cm^−2^ loading of Pt [36]. The annealing-induced transformation of the PtNi/C to PtNi–O/C structure is shown in Fig. 4B(a). As shown in Fig. 4B(b), the mass activity at overpotential of 70 mV versus reversible hydrogen electrode (RHE), the PtNi–O/C present the highest activity of 7.23 mA/µg_pt_ in comparison with those obtained for PtNi (5.35 mA/µg_pt_) and commercial Pt/C (0.92 mA/µg_pt_).

**Fig. 5:**
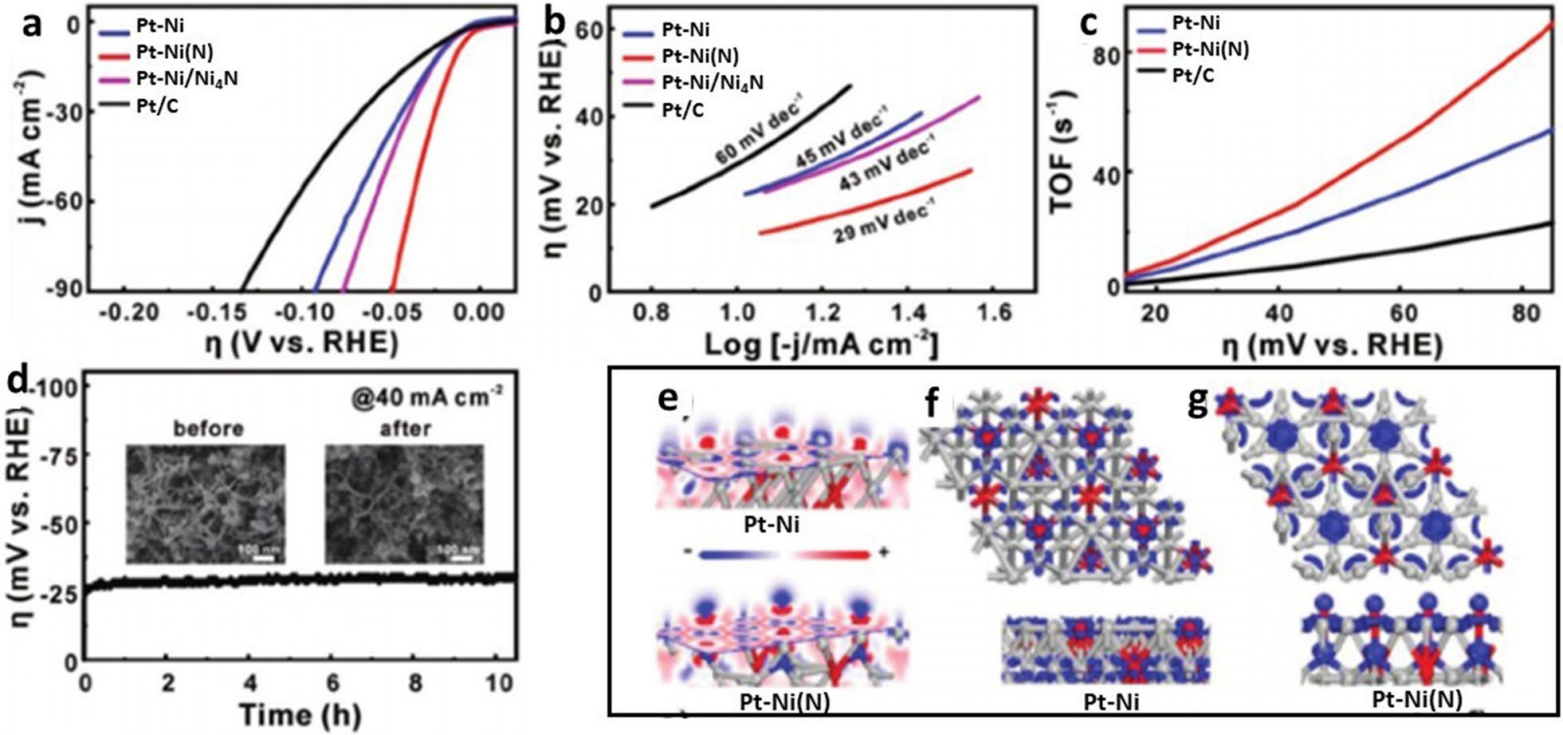
**a**–**d** HER performance, **a** LSV curves comparing different catalysts in 1.0 M KOH with IR correction, **b** Tafel plots, **c** TOF curves, **d** Chronopotentiometric curve of Pt–Ni(N) NWs. Insets: SEM images of Pt–Ni(N) NWs before and after the stability test. **e**–**g** DFT calculation results: **e** electron density difference for slices of Pt–Ni and Pt–Ni(N), **f**, **g** top-view and side-view of orbitals above the Fermi level for Pt–Ni and Pt–Ni(N) (Figure reprinted with permission from Ref. [37])

Doping of Pt-based materials with different metal components is another effective way to improve the catalytic ability for HER while reducing the use of Pt. Wang et al. [37] reported a catalyst derived from N modified PtNi nanowires, which was shown to boost water dissociation kinetics by N-induced orbital tuning of the catalyst, delivering an ultralow overpotential of 13 mV at 10 mA cm^−2^ in alkaline media. Figure 5a–d shows the HER catalytic performance of PtNi(N) NWs in alkaline media in terms of linear sweep voltammogram (LSV) curves (a) for Pt–Ni, Pt–Ni(N), Pt–Ni/Ni_4_N, and commercial Pt/C. Pt–Ni(N) NWs exhibit the lowest overpotential of 13 mV at a current density of 10 mA cm^−2^. The Tafel slopes, as shown in Fig. 5b, provide information for probing the rate-determining steps in the alkaline HER process. The Tafel slope for Pt-Ni (N) reaches 29 mV dec^−1^, suggesting that the Volmer step is not the rate-determining step for Pt–Ni(N) in the alkaline media. Further, the analysis of the TOF data provides information for unraveling the intrinsic activity of catalysts, as shown in Fig. 5c. Pt–Ni(N) exhibits a higher TOF than that for Pt/C and Pt–Ni NWs under various potentials. Lastly, as shown in Fig. 5d, the durability test of Pt–Ni(N) by chronopotentiometry shows that there is no obvious potential change after 10 h at the current density of 40 mA cm^−2^. Density function theory (DFT) calculation (Fig. 5e–g) was used to study the modulation essence of nitrogen dopants in the PtNi nanowire. The analysis of the surface electron density difference (Fig. 5e) shows that the introduction of N could decrease the electron density around Ni site by strong interaction between N and Ni. The orbital analysis (Fig. 5f, g) further confirms the strong Ni–N coupling generated in the direction perpendicular to empty dz^2^ with an optimal orientation for water dissociation and activation.

#### Non-noble metal based electrocatalysts

**Fig. 6:**
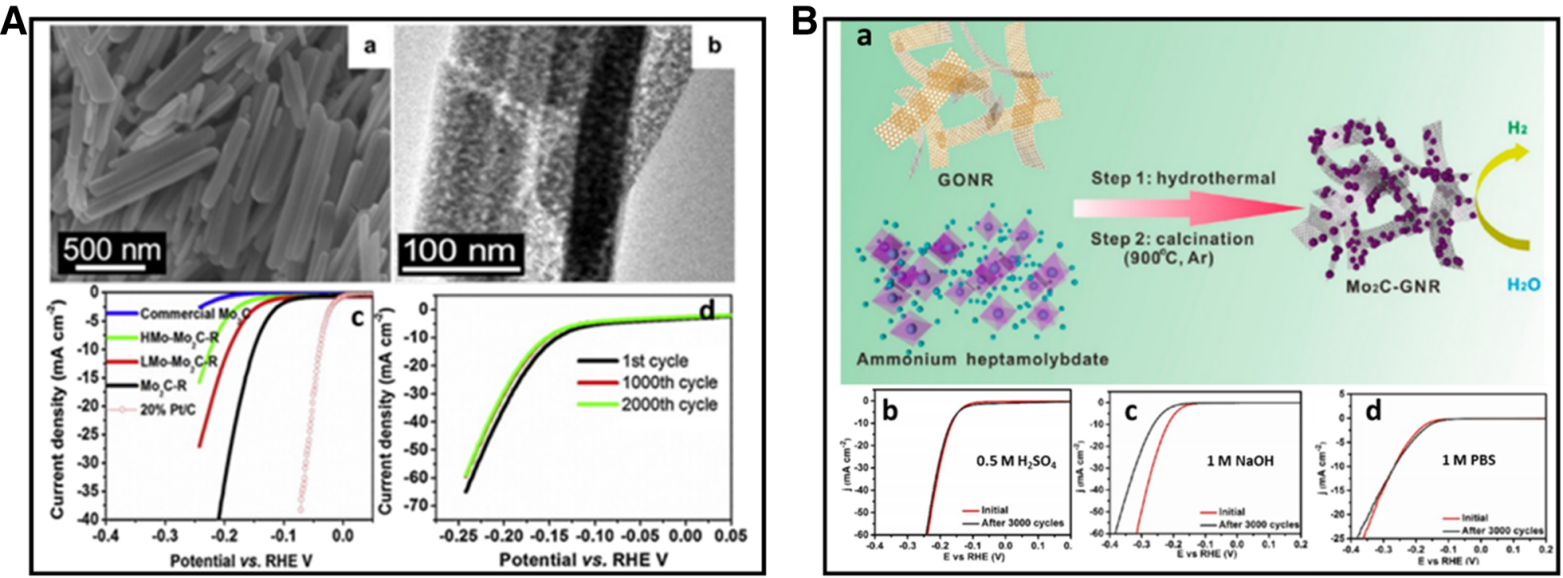
**A** (a) FESEM image of Mo_2_C-R, (b) SEM image of Mo_2_C-R, (c) polarization curves in 0.5 M H_2_SO_4_, and (d) stability test of Mo_2_C-R in 0.5 M H_2_SO_4_ (Figure reprinted with permission from Ref. [38]). **B** (a) Preparation of molybdenum carbide (Mo_2_C) nanoparticles anchored on graphene nanoribbons (GNRs), and (b–d) activity and durability of Mo_2_C-GNR in acidic, basic, and neutral media, respectively (Figure reprinted with permission from Ref. [39])

*Transition metal carbides (TMCs)* TMCs have received extensive interest in the development of non-noble metal based electrocatalysts. For example, Mo_2_C and WC are shown to exhibit high catalytic activity toward HER. In addition to the high electrical conductivity, their properties of hydrogen adsorption and d-band electronic density state (similar to that of Pt) exhibit an optimal combination that is considered to be the main factor for the observed high HER activity. Back to 1973, Levy and Boudart first discovered that tungsten carbide possessed d-band electronic density states similar to that of Pt species and thus exhibited platinum-like catalytic behavior [49]. Further, Chen et al. employed DFT calculation to investigate the physical, chemical, and electronic structure properties of a series of transition metal carbides. Their results show that the incorporation of carbon atoms into the lattice interstitials afford them with d-band electronic density states similar to the Pt benchmark [50]. The theoretical result was first supported by experimental data in 2012. Hu et al. [51] reported that commercially available molybdenum carbide microparticles (com-Mo_2_C) possess good HER catalytic activity in both acidic and alkaline media. However, there is a relatively large overpotential (190–230 mV) to achieve a cathodic current of 10 mA cm^−2^. Inspired by the pioneering studies, researchers pursued different approaches for the optimization of Mo_2_C catalyst by nanoengineering the materials to expose more active sites. Wang et al. [38] have successfully synthesized Mo_2_C nanorods with a porous structure by facile carburization of anilinium molybdate in hydrogen, as shown in Fig. 6A. The nanorod morphologies with a smooth surface and a porous structure are revealed by field emission scanning electron microscope (FE-SEM) and transmission electron microscopy (TEM) images (Fig. 6A(a, b)). Enhanced HER electrocatalytic performance is shown for Mo_2_C nanorod catalyst. Figure 6A(c) shows the linear scan voltammetry (LSV) results in 0.5 M H_2_SO_4_ which exhibits the Mo_2_C nanorods has a better activity than the commercial Mo_2_C. The stability test of the Mo_2_C is also shown in Fig. 6A(d). The results show that the activity is stable after 2000 cycles, demonstrating an excellent cycling life. The Mo_2_C nanorod is also studied in alkaline media, showing an advantage in performance over commercial Mo_2_C in 1 M KOH. The Mo_2_C nanorods display a competitive performance for HER in both acidic and alkaline media, which stem from the high conductivity and well-defined, and porous morphology. The catalytic activity could be further improved by loading Ni nanoparticles. The deposition of molybdenum carbides on carbon-based materials is another method to further improve the performance of HER as an effective hybrid nano-electrocatalyst. Liu et al. [39] prepared molybdenum carbide (Mo_2_C) nanoparticles anchored on graphene nanoribbons (GNRs) by in situ growth of carbides on GNR template through hydrothermal synthesis and subsequent high-temperature calcination, as shown in Fig. 6B(a). The Mo_2_C-GNR hybrid exhibits outstanding electrocatalytic activity and durability in all the acidic, basic, and neutral media (Fig. 6B(b–d)). The use of GNRs as templates for the in situ growth of carbides is an intriguing approach since the interconnected GNR network structure may provide multiple conductive pathways for fast electron transport and large accessible surface area with increased exposed active sites, leading thus to enhanced catalytic activity in all the acidic, basic, and neutral media.

**Fig. 7:**
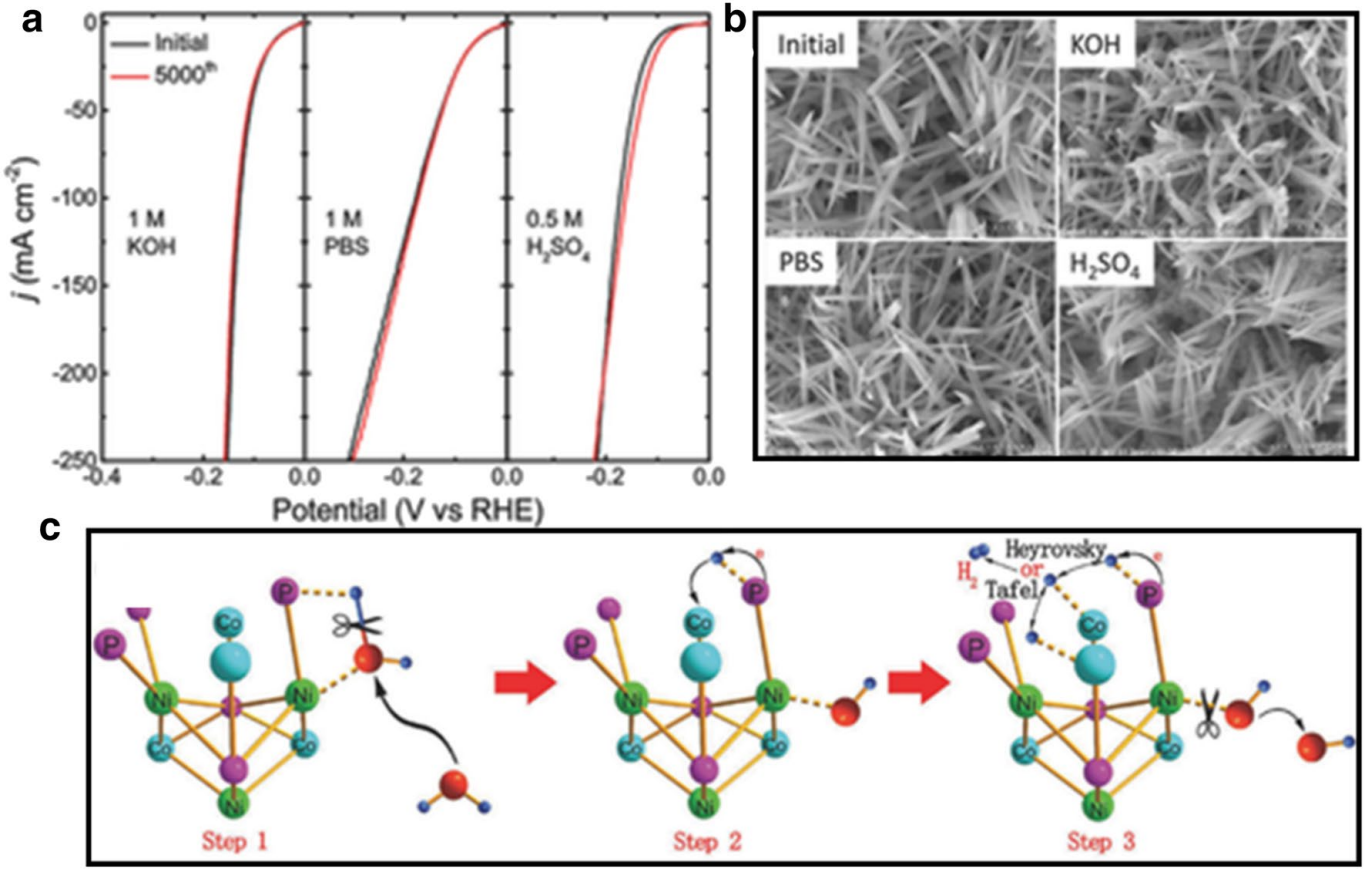
NiCo_2_P_X_ catalyst: **a** HER performance in 1 M KOH and PBS and 0.5 M H_2_SO_4_, **b** SEM images before and after HER long-term stability test in different media, **c** schematic illustration of water dissociation over NiCo_2_P_X_ in alkaline solution (Figure reprinted with permission from Ref. [41])

*Transition metal phosphides* The exploration of transition metal phosphides (TMP) is one of the rapidly growing areas in developing electrocatalysts with high catalytic activity and stability in both acid and basic (pH universal). It has been proposed that P atom plays a significant role in TMP due to the excellent conductivity and unique electronic structure. The discovery of Ni_2_P catalysts as one of the best practical catalysts for HER dates back to 2005. Liu and Rodriguez investigated a series of electrocatalysts by density functional theory (DFT) [52]. The results show the HER activity in the trending of [NiFe] hydrogenase > [Ni(PNP)_2_]^2+^ > Ni_2_P (001) > [Ni(PS_3_*)(CO)]^1−^ > Pt > Ni. The reason that Ni_2_P displays a superior activity over the bulk Pt and Ni is because hydrogen formed in the HER process strongly bonds on metal (Pt, Ni) hollow site. The strong bonding would lead to an increased desorption energy for the hydrogen species from the metal surface. The presence of P atoms dilutes the concentration of highly active Ni sites, leading to a moderate bonding to the intermediates and products, i.e., the so-called “ensemble effect”. The first direct experimental evidence supporting the catalytic synergy was provided by Popczun et al., who demonstrated that Ni_2_P nanoparticle deposited onto a Ti foil substrate for excellent HER activity, showing an exchange density of 3.3 × 10^–5^ mA cm^−2^ and a Tafel slope of 46 m V dec^−1^ [40]. However, the stability of the Ni_2_P/Ti electrode was not satisfactory especially in alkaline electrolyte. Further investigation was carried by Hu et al. [41] by developing a bimetallic-structured phosphide electrocatalyst, NiCo_2_P_x_. This catalyst shows excellent durability and long-term stability in different electrolytes, an efficient pH-universal catalyst performance for HER. The self-supported NiCo_2_P_x_ nanowire arrays were fabricated on commercial carbon felt (CF) by a wet chemical-hydrothermal route combined with subsequent in situ phosphorization reaction. Figure 7a shows that NiCo_2_P_x_ exhibits excellent HER performance in acidic, alkaline, and neutral media. In the alkaline electrolyte, NiCo_2_P_x_ has the lowest overpotential of 58 mV at a current density of 10 mA cm^−2^ compared with CoP_x_ (94 mV), NiP_x_ (180 mV), and commercial Pt (70 mV). Figure 7b shows the morphology of NiCo_2_P_x_ after the long term HER activity test, demonstrating that the catalyst structure of NiCo_2_P_x_ is still well-preserved after 5000 cycles under different conditions. A synergistic effect is proposed for NiCo_2_P_X_ in alkaline electrolyte, as shown in Fig. 7c. There are two important interactions: the interaction between the under-coordinated metal center (M^δ+^, M = Ni, Co) and O atom, and the interaction between the dangling P atom (P^δ−^) and H atom. The combination of these two interactions weakens the H–OH bond, leading to water molecule dissociate into H atom and OH^−^. The H atom is then transferred onto a nearby vacant metal site anchored as adsorbed H, and the adsorbed H atoms combine to form molecular H_2_.

**Fig. 8:**
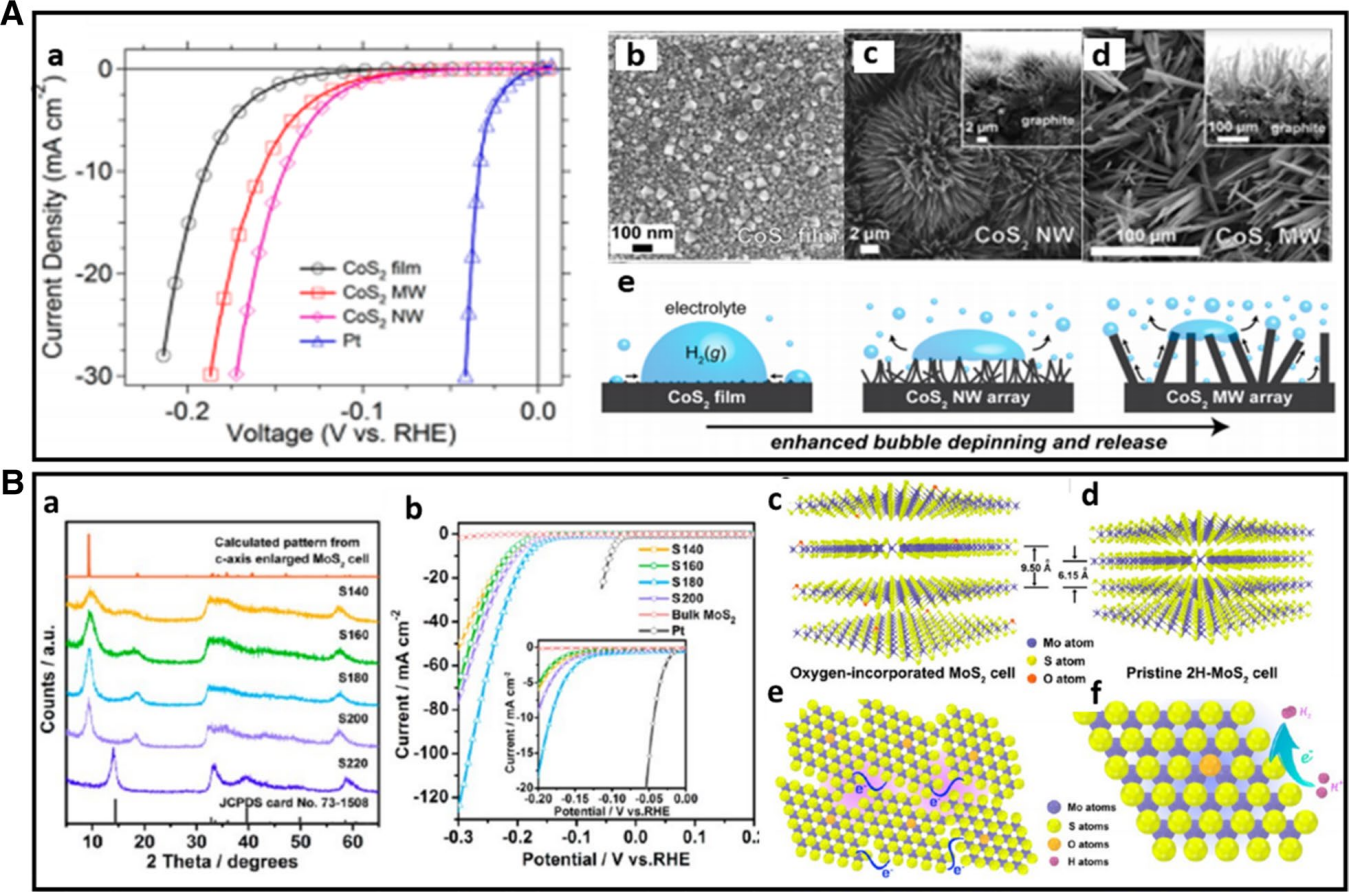
**A** (a) Polarization curves of CoS_2_ film, MW array, and NW array electrode for HER, (b–d) SEM images of CoS_2_ film, NW array, and MW array on graphite, (e) schematic depictions of CoS_2_ film, CoS_2_ NWs, and MWs surface along with the produced H_2_(g) bubbles (Figure reprinted with permission from Ref. [43]). **B** (a) XRD pattern of the products obtained at various temperatures, (b) HER performance of the catalysts with different oxygen-incorporated MoS_2_ ultrathin nanosheets. (c, d) Structural models of the oxygen-incorporated MoS_2_ with enlarged interlayer spacing and the pristine 2H–MoS_2_. (e, f) Schematic representation of the disordered structure in oxygen-incorporated MoS_2_ ultrathin nanosheets. The blue lines represent the fast electron transport between the quasi-periodically aligned nanodomains; the purple shadings indicate the enrichment effect of active sites arising from the disordered structure (Figure reprinted with permission from Ref. [44])

*Transition metal chalcogenides (sulphides and selenides)* By DFT calculation in 2005, Nørskov et al. [53] showed that the free energy of atomic hydrogen bonding to the MoS_2_ edge is close to that of Pt. This finding identifies MoS_2_ as a promising electrocatalyst for HER. To further identify the actual active site of MoS_2_ structure, Chorkendorff et al. [54] prepared triangular MoS_2_ single crystals with different sizes on Au(111) substrate. They demonstrated that the electrocatalytic HER activity is linearly dependent on the number of edge sites of the MoS_2_ catalyst. The edge sites of the MoS_2_ are highly catalytically active sites. Inspired by this understanding, various strategies have been proposed to expose the active sites to improve HER activity, including nanostructure tailoring and morphology tuning. For example, Xie et al. [42] reported a strategy to engineer the defects in MoS_2_ ultrathin nanosheets, which was shown to dramatically improve the electrocatalytic HER performance of MoS_2_. This high activity was attributed to additional active edge sites of the rich-defect structure synthesized by partial cracking of the catalytically inert basal plane. In another study, Jin et al. [43] successfully synthesized CoS_2_ with controllable film, microwire (MW), and nanowire (NW) morphologies (Fig. 8A(b–d)). They systematically studied their structures, activities, and stabilities and demonstrated the morphology-dependent enhancement of both activity and stability. Among the three different morphologies, CoS_2_ NWs show the highest HER catalytic performance and stability (Fig. 8A(a)), which is attributed to the high effective electrode surface area and high speed to release of evolved gas bubbles from the electrode surface (Fig. 8A(e)). In addition to engineering active sites, considerable efforts have been devoted to engineering the electronic conductivity of metal chalcogenides to optimize the HER electrocatalytic activity. Heteroatom doping is an effective method to enhance HER activity. Xie et al. [44] found that incorporation of oxygen atom and controllable disorder engineering can effectively regulate the electronic structure of MoS_2_ ultrathin nanosheets, leading to the enhancement of conductivity and HER activity. As shown in Fig. 8B(a) with a series of XRD patterns for the catalysts obtained at various temperatures, the MoS_2_ ultrathin nanosheets obtained at high temperature (220 °C) exhibit rich defects. When the synthesis temperature decreases to 200 °C and below, two new peaks emerge at the low-diffraction angle region, indicating the presence of a new lamellar structure with an enlarged interlayer spacing of 9.5 Å compared with that of 6.15 Å in pristine 2H–MoS_2_. The proposed structure models are shown in Fig. 8B(c, d). HRTEM images and corresponding FFT patterns of the MoS_2_ structures from the different temperatures reveal a controllable disorder engineering with the degree of disorder depending on the temperature. The electrochemical measurements of the oxygen-incorporate MoS_2_ ultrathin nanosheets with different degrees of disorder were performed in Fig. 8B(b). The catalyst labeled as S180 exhibits the lowest potential (120 mV), suggesting the superior HER activity. Figure 8B(e, f) shows schematic representation of the disordered structure in oxygen-incorporated MoS_2_ ultrathin nanosheets. There is a fast electron transport between the quasi-periodically aligned nanodomains (Fig. 8B(e)). The enrichment effect of active sites is derived from the disordered structure (purple shading). The excellent HER electrocatalytic activity is attributed to the disordered structure which offers large amounts of unsaturated sulfur atoms as active sites for HER and provides quasiperiodic arrangement of nanodomains for a fast interdomain electron transport.

## Electrocatalysts for oxygen evolution reaction (OER)

As stated earlier, OER is the other key half-reaction in water-splitting reaction. This reaction occurs at the anode and involves a four-electron transfer process which requires a remarkably high overpotential compared to HER. OER is known to be the major bottleneck in improving the overall efficiency of electrochemical water splitting. Therefore, it is imperative to seek highly efficient OER catalysts that can effectively reduce the kinetic limitation. Significant progress has been made in the recent development of the understanding of OER mechanism for the rational design of OER electrocatalysts. It is widely accepted that OER can proceed through two different mechanisms conventional adsorbate evolution (AEM) and lattice oxygen-mediated mechanism (LOM), which are discussed in two of the following subsections.

### Reaction steps in OER—adsorbate evolution mechanism (AEM)

**Fig. 9:**
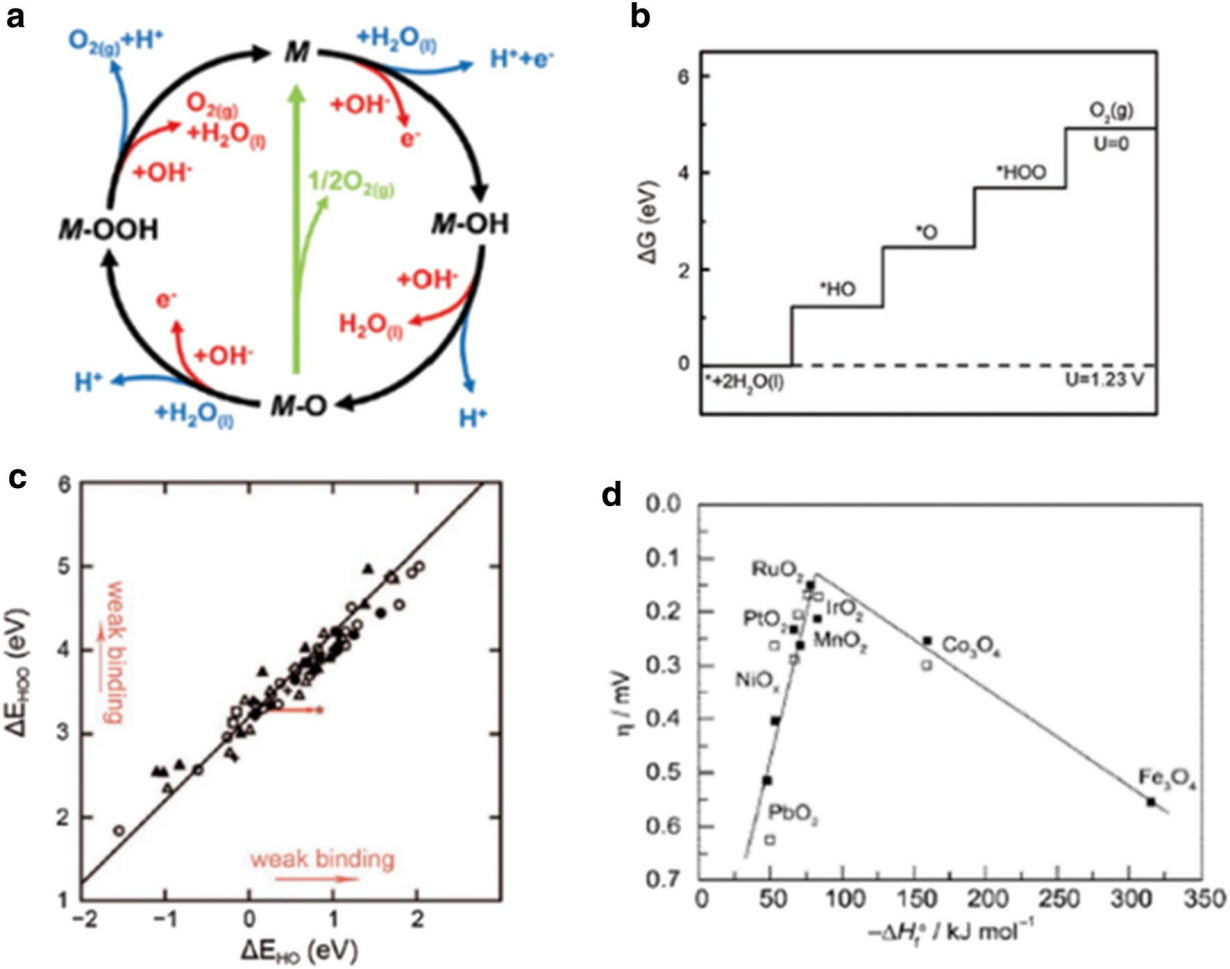
**a** OER mechanisms in acidic (blue line) and alkaline (red line) electrolytes (Figure reprinted with permission from Ref. [5]). **b** Free energy diagram at U = 0 and U = 1.23 V for OER on the ideal catalyst (Figure reprinted with permission from Ref. [55]). **c** Relationship between the adsorption energies of *OOH and *OH on a series of oxide OER catalysts (Figure reprinted with permission from Ref. [56]). **d** Volcano-shaped relationship between OER activities on metal oxide surfaces vs. enthalpy for the transition metal oxides in acidic (black square) and basic solution (white square) (Figure reprinted with permission from Ref. [57])

For OER, adsorbate evolution mechanism (AEM) have conventionally been used to describe the various reaction steps. In AEM, the reaction typically involves four concerted proton and electron transfers with the metal centers as active site (M), producing oxygen molecules from water in acidic and alkaline media (Fig. 9a) [5]. The reaction pathway of alkaline OER (in red line) includes the following steps (3a–3d):

3a $$ {\text{OH}}^{ - } + {\text{M}} \to {\text{M}} - {\text{OH}} + {\text{e}}^{ - } , $$

3b $$ {\text{M}} - {\text{OH }} + {\text{ OH}}^{ - } \to {\text{ M}} - {\text{O }} + {\text{ H}}_{{2}} {\text{O }} + {\text{ e}}^{ - } , $$

3c $$ {\text{M}} - {\text{O}} + {\text{OH}}^{ - } \to {\text{M}} - {\text{OOH}} + {\text{e}}^{ - } /\;2{\text{M}} - {\text{O}} \to 2{\text{M}} + {\text{O}}_{2} + 2{\text{e}}^{ - } , $$

3d $$ {\text{M}} - {\text{OOH}} + {\text{OH}}^{ - } \to {\text{O}}_{2} + {\text{H}}_{2} {\text{O}} + {\text{e}}^{ - } + {\text{M}}. $$

Firstly, hydroxide anions are adsorbed on the metal active site to form M–OH. Then M–OH forms M–O after deprotonation. Thereafter, there are two different pathways to form O_2_ molecules. One way is that M–O reacts with OH^−^ to form M–OOH intermediate, producing O_2_ through the deprotonation of M–OOH with the regeneration of the active site. The other way, as illustrated by the green route as in Fig. 9a, involves two M–O species being combined and converted into O_2_ along with the regeneration of the M active site, which is considered to have a large activation barrier. For the mechanism of acidic OER, the common consensus is that the same intermediates such as M–OH, M–O, and M–OOH are involved. For electrocatalysis of OER, a detailed understanding of the binding strength of the reaction intermediates on the electrode surface is crucial for the enhancement of the overall OER performance because the binding strength is a key parameter governing the reaction overpotential.

As shown by the ideal free energy diagram of the different reaction steps in OER in Fig. 9b, there is no overpotential needed for OER to occur if the free energy gap for each of the elementary steps would remain the same at 1.23 eV [55]. However, this ideal case is almost impossible to achieve. The OER overpotential is determined by the rate-determining step (RDS) which comes from the step with the most positive reaction free energies (ΔG) in the four steps. Based on a database of diverse oxide catalyst model, there is a scaling relationship (linear correlation) established in the AEM in terms of the binding energies of these intermediates (M–OH, M–OOH, and M–O), as shown in Fig. 9c. The binding energies of the adsorbed M–OH and M–OOH exhibit a constant difference of 3.2 eV(ΔG_HOO*_ − ΔG_HO*_). This is because both HOO* and HO* bind with catalyst surface through similar adsorption configurations with an oxygen atom via a single bond [56]. According to the scaling relation, there is a minimum theoretical overpotential of 0.37 eV((3.2 − 1.23*2 eV)/2) which represents the difference between the constant difference in binding energy (3.2 eV) and the ideal value of 2.46 eV.

Moreover, since the second (the formation of M–O) and third steps (the formation of M–OOH) are considered as the RDS in most OER catalyst, the difference between ΔG_O*_ and ΔG_HO*_ is used as a universal descriptor to predict their OER activity. This is represented by the Sabatier’s volcano-shaped relationship which has been traditionally used to explain the OER activity trends on metal oxides in both acidic and alkaline media. The best catalysts in terms of the lowest theoretical overpotential for OER are IrO_2_ and RuO_2_, which exhibit optimal binding strength on the catalyst surface, i.e., neither too strong nor too weak, as shown in Fig. 9d [57].

### OER electrocatalysts

Table 2 lists some selected OER electrocatalysts in terms of their performance descriptors, which will be further discussed in later sections under different reaction conditions. There are two main types of OER electrocatalysts, noble-metal based electrocatalysts, and non-noble metal based electrocatalysts. For the noble-metal based electrocatalysts, Ru and Ir-based catalysts are considered as the state-of-the-art electrocatalyst for OER, especially in the acidic electrolyte, which has a larger dissolution resistance compared with other metals. To reduce the high price, improve the electrocatalyst activity, stability, and even enforce the dissolution resistance in acidic media, there are several strategies to engineer and optimize the catalyst composition, structure, and morphology. Other than Ir and Ru, other noble metals such as Rh, Au, Pt, and Pd-based catalysts have also been developed as bi- or tri-functional electrocatalysts which show promising performance for OER, HER, and oxygen reduction reaction (ORR). For the non-noble metal based catalysts, the earth-abundant oxide and (oxy)hydroxide electrocatalysts have received a great deal of interest for OER, especially Ni–Fe based oxide and (oxy)hydroxide, some of which are the most common OER catalyst being employed in the industry-scale development. In the following subsections, we will highlight different strategies that are applied to further improve the activity and stability of NiFe-based electrocatalysts.

**Table 2:** Summary of the OER performance of the reported electrocatalysts

Catalysts	Electrolyte	η (mV)	*I* (mA cm^−2^)	Tafel slope (mV dec^−1^)	Stability	Refs.
Cu-doped RuO_2_	0.5 M H_2_SO_4_	188	10	43.96	10,000 cycles	[58]
IrNi NPNW	0.1 M HClO_4_	283	10	56.7	200 min	[59]
IrCo NPNW	0.1 M HClO_4_	295	10	60.3		
IrFe NPNW	0.1 M HClO_4_	302	10	68.5		
IrNiCu DNF	0.1 M HClO_4_	300	10	48	2500 cycles	[60]
IrO_2_ NN	1 M H_2_SO_4_	313	10	57	200 h	[61]
Au_40_Co_60_ NPs	0.1 M KOH	175	10	65	1 h	[62]
G-FeCoW	1 M KOH	191	10		500 h	[63]
Plasma-engraved Co_3_O_4_	0.1 M KOH	153	10	68	2000 cycles	[64]
NiFe-LDH NPs	0.1 M KOH	151	30	50	10 h	[65]
Ni_0.83_Fe_0.17_(OH)_2_	1 M KOH	245	10	61	10 h	[66]
Ni_x_Fe_1−x_Se_2_-DO	1 M KOH	195	10	28	24 h	[67]

#### Noble-metal based electrocatalysts

Noble metal and metal oxide electrocatalysts have long been considered as the most powerful electrode materials in OER. Examples include RuO_2_ and IrO_2_, which are usually considered as the state-of-the-art electrocatalysts for OER. However, the high price and serious dissolution of RuO_2_ and IrO_2_ are the major concerns, which bring great attention to the modification of the catalysts to enable composition and structure/morphology optimization. Several strategies have been proposed to improve the electrocatalyst activity and stability and reduce the high cost.

**Fig. 10:**
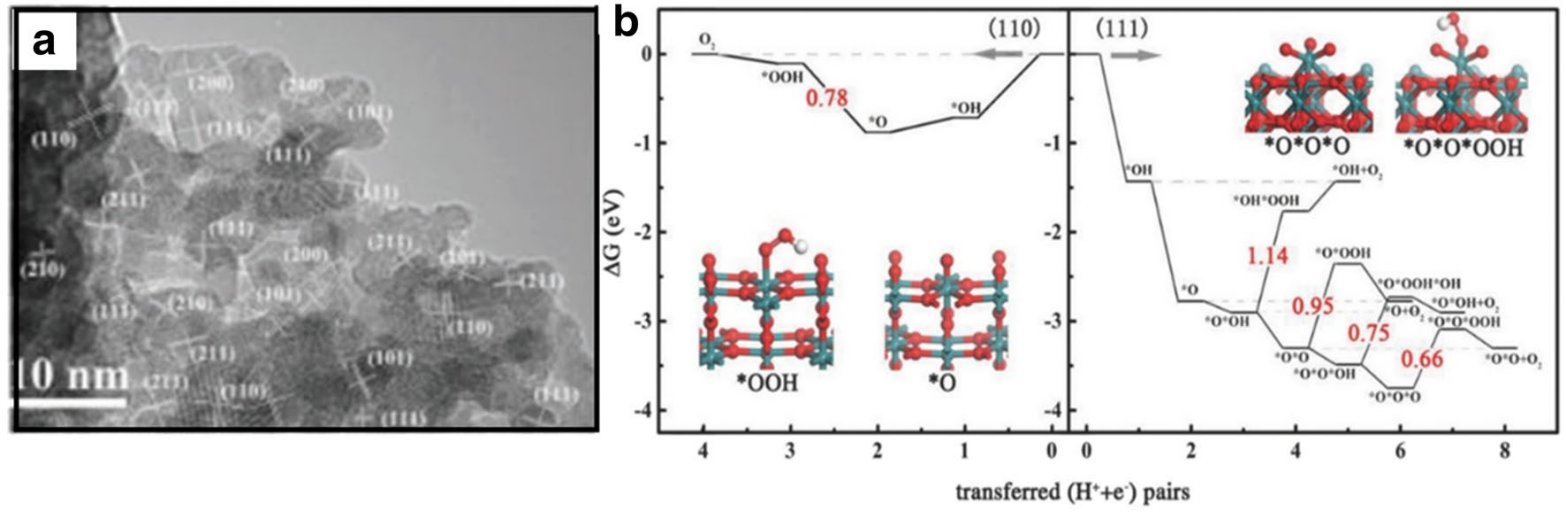
**a** High-resolution TEM (HRTEM) of Cu-doped RuO_2_ with high-index surface facets being indicated. **b** Free energy profile of OER on (110) and (111) surfaces (Figure reprinted with permission from Ref. [58])

Heteroatom doping for tuning the composition of IrO_2_-based OER electrocatalysts has generated a great deal of interest. However, different guest atoms generated distinct energy domains for the host system. Chen et al. prepared Cu-doped RuO_2_ with hollow porous polyhedral morphology composed of ultrasmall nanocrystals by thermal decomposition of Ru-exchanged metal–organic framework (MOF) derivatives. The catalyst displays a remarkable OER performance with a low overpotential of 188 mV at 10 mA cm^−2^ in acidic electrolyte and excellent stability in durability testing for 10,000 cycles [58]. High-resolution TEM (HRTEM) and XRD data reveal the Cu is incorporated into the RuO_2_ lattice to form Cu-doped RuO_2_ rutile phase, as shown by the high-index surface facets in Fig. 10a. The high OER activity is attributed to the high-index surface which contains highly under-coordinated Ru (CN = 3) sites that can effectively reduce the OER overpotential, as shown by DFT calculation in Fig. 10b. The formation of *OOH on RuO_2_(110) (Fig. 10b) was found to be the RDS, with an energy barrier of 0.78 eV. For other high-index facets of the RuO_2_(111) surface, only 0.66 eV is needed to overcome the energy barrier, which contributes to the decreased overpotential of 120 mV. The Cu-dopant RuO_2_ not only can induce the formation of unsaturated Ru sites by Cu dopant generated O vacancies on the surface, but also can modulate the electronic structure which exhibits a broad binding region closer to the Fermi level of p-band center, leading to the enhancement of the OER activity.

**Fig. 11:**
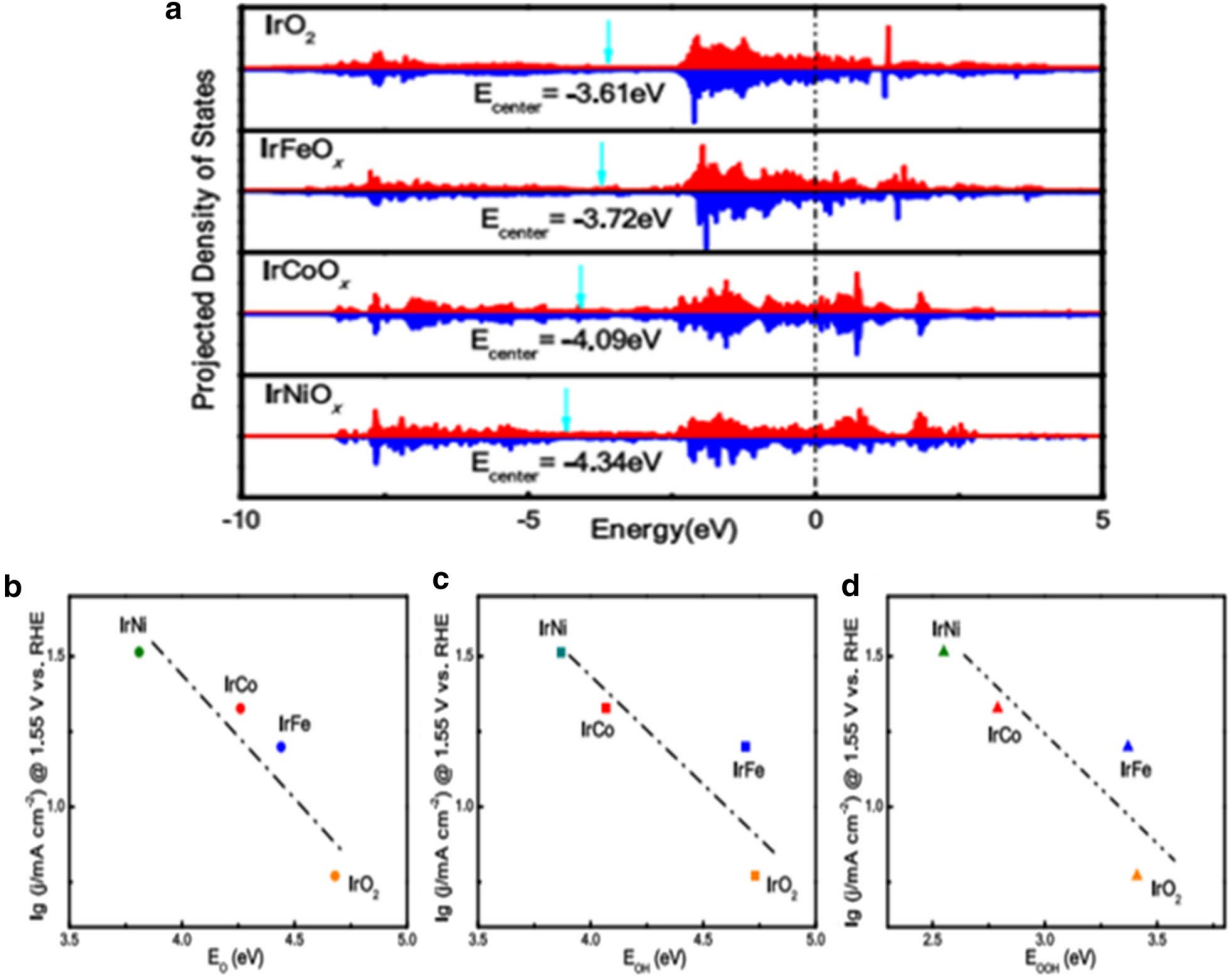
**a** Projected DOS of Ir 5d bands of IrO_2_ and IrMOx model. **b**–**d** Relationship between the activity (indicated by the logarithm of the as-measured current density at 1.55 V vs. RHE) and the binding energies of different intermediate species: **b** O, **c** OH, and **d** OOH (Figure reprinted with permission from Ref. [59])

Alloying Ru or Ir with other transition metals is an intriguing strategy to engineer the OER catalysts, which can effectively modify the electronic structure and optimize the adsorption energy of the reaction intermediates. Zhang et al. [59] designed a class of IrM (M=Ni, Co, Fe) catalysts with a unique network structure composed of intertwining nonporous nanowires by a eutectic-directed self-templating strategy. The results show a transition-metal dependent feature. Compared with IrFe NWs and IrCo NWs, IrNi NWs exhibit the best OER activity with the lowest overpotential of 283 mV at 10 mA cm^−2^. The high activity of IrNi NW was explained by DFT calculation results (Fig. 11). During the OER process, Ir and M are oxidized at a high potential to form IrMO_x._ The d band centers for IrO_2_, IrFeO_x_, IrCoO_x_ and IrNiO_x_ are − 3.61, − 3.72, − 4.09, and − 4.34 eV, respectively (Fig. 11a). There is a negative shift of density of state (DOS). The downshift for Ir’s d band center indicates that the d-band electrons distribute away from the Fermi level, which is caused by the ligand effect after alloying. As shown in Fig. 11b–d, the OER activity strongly depends on the binding energies of O, OH, and OOH species. Consequently, the introduction of 3d transition metals can shift down the Ir d-band center and weaken the adsorption strength of the reaction intermediate species, leading to the exhibition of the 3d transition-metal dependent OER activity.

**Fig. 12:**
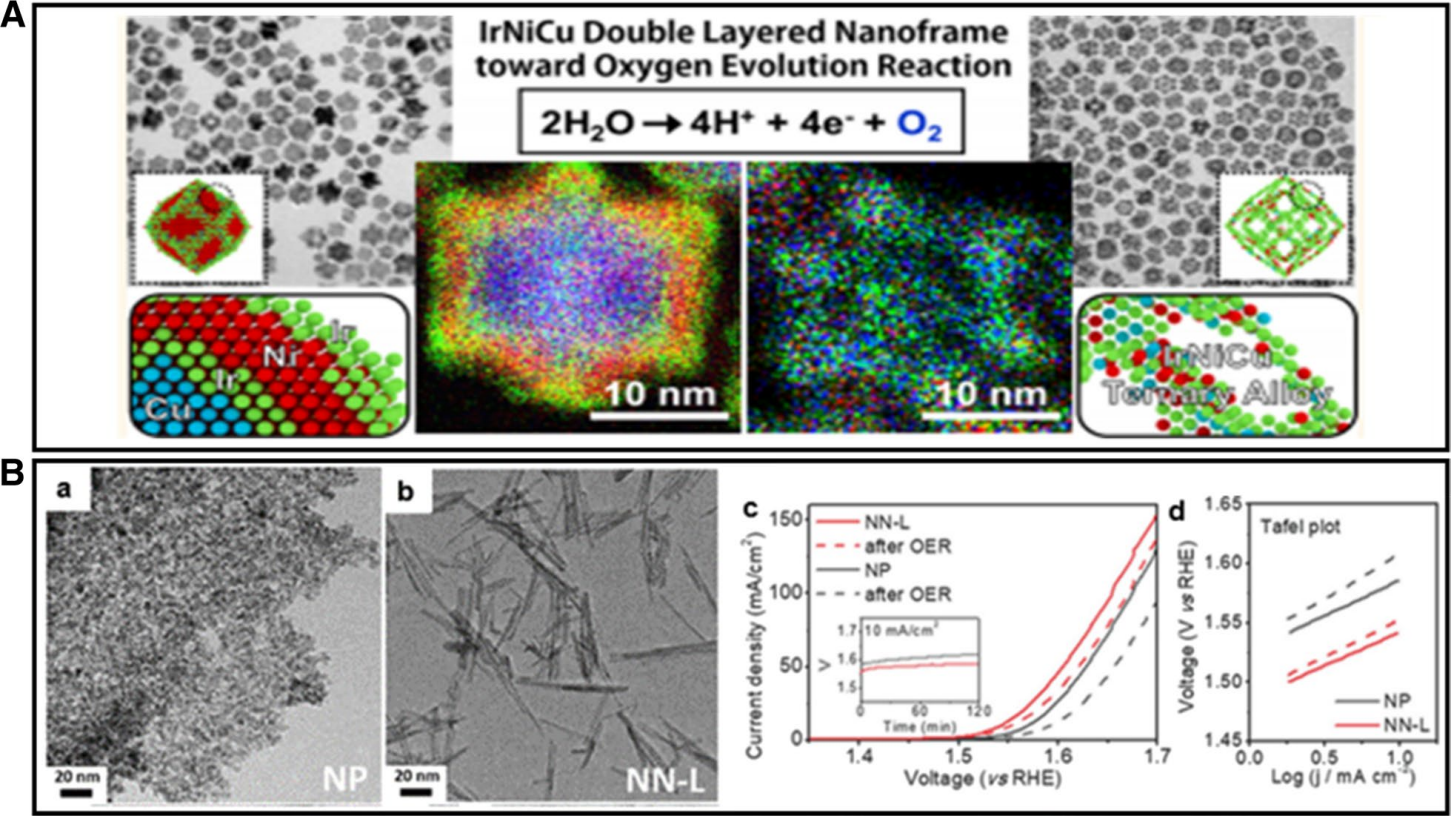
**A** The HAADF-STEM, TEM, HRTEM, and elemental mapping of desired IrNiCu DNF structure (Figure reprinted with permission from Ref. [60]). **B** (a, b) TEM images of IrO_2_ nanoparticles and IrO_2_ nanoneedles, (c, d) OER performance before and after 2 h galvanostatic operation, (c) LSV curves of IrO_2_ NN and unshaped nanoparticles, (d) Tafel slope (Figure reprinted with permission from Ref. [61])

Surface structure modifications play an extremely important role in exposing catalytically active sites and taking advantage of the interfacial effect. Morphology regulation, as one aspect of the surface structure modification, has received increasing attention. For example, hollow nanoparticles such as nanocages, nanoshells, and nanoframes have been proved effective for the enhanced catalytic activity, reflecting their most open structure with increased catalytically active sites. Lee et al. [60] demonstrated that an Ir-based multimetallic double-layered nanoframe (DNF) electrocatalyst can be formed by a simple one-step synthesis. The HAADF-STEM, TEM, HRTEM, and elemental mapping data revealed that the desired IrNiCu DNF structure preserves a rhombic dodecahedral morphology after strong acid etching and has a uniform component distribution in the entire DNF structure (ternary alloy), as shown in Fig. 12A. The excellent electrocatalytic activity and durability of the IrNiCu catalyst for OER are attributed to a frame structure that prevents the coarsening and agglomeration of particles and in situ formation of robust rutile IrO_2_ phase during OER process. Morphology control is also shown to affect the electrical conductivity of the catalyst, which improves the OER electrocatalytic activity. Lee et al. synthesized ultrathin IrO_2_ nanoneedles (Fig. 12B(a, b)) by a scalable molten salt method which show a better OER activity and stability compared with IrO_2_ nanoparticle. As shown by the electrochemical performance of IrO_2_ NPs and IrO_2_ nanoneedles for OER in Fig. 12B(c, d), the IrO_2_ nanoneedles exhibit better activity and stability than those for IrO_2_ NPs. In comparison with the electrical conductivity, 25.9 S cm^−1^, for IrO_2_ unshaped nanoparticles, the conductivity for ultrathin IrO_2_ nanoneedles is 318.3 S cm^−1^, suggested the shape of the catalyst plays an important role in the electron transfer induced high activity for OER [61].

**Fig. 13:**
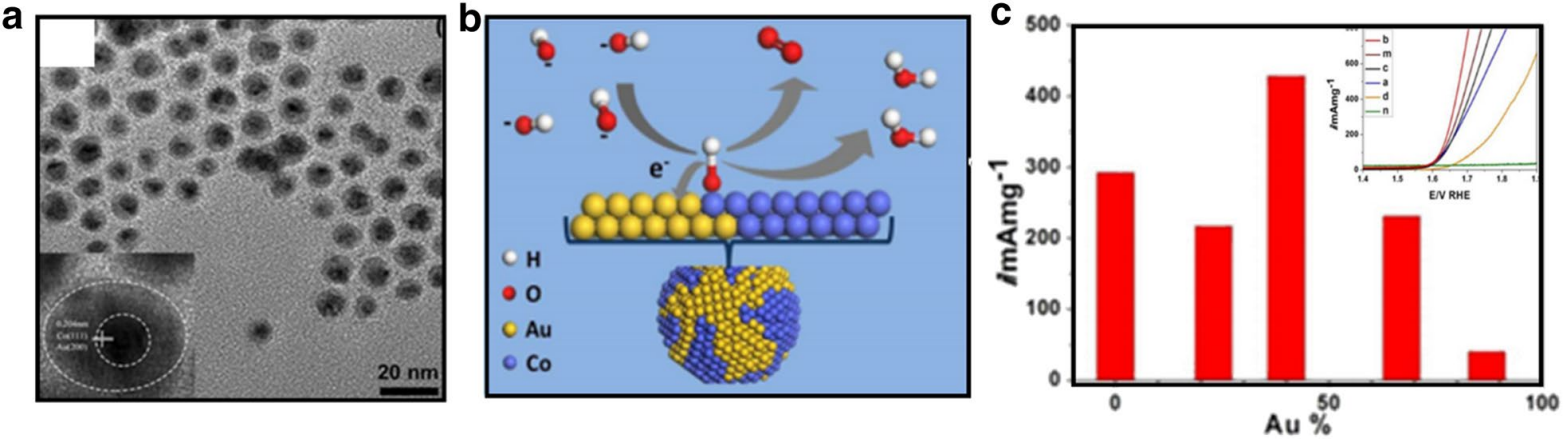
**a** TEM images of AuCo core–shell nanoparticles (inset: high-magnification TEM showing the lattice fringes corresponding to fcc Au and fcc Co), **b** schematic illustration of OER on the partially phase segregated AuCo nanoparticles, **c** comparison of catalytic activities at 1.67 V in 0.1 M KOH. Inset: polarization curves of (a) Au_23_Co_77_/C, (b) Au_40_Co_60_/C, (c) Au_71_Co_29_/C, (d) Au_95_Co_5_/C, (m) CoO_x_/C, (n) Au/C in 0.1 M KOH (Figure reprinted with permission from Ref. [62])

Nobel metals other than Ir and Ru, such as Rh, Au, Pt, and Pd, have also been emerging as viable OER electrocatalysts. The design of Pt, Pd, Ru, and Au catalysts involves the construction of bi- or tri-functional electrocatalysts for OER, oxygen reduction reaction (ORR), and hydrogen evolution reaction (HER). Because Rh, Pt, Au, and Pd have smaller dissolution resistances than Ir and Ru in an acidic electrolyte with a large overpotential, the evaluation of their OER behaviors has usually been conducted in alkaline solution. The ability to control the morphology and composition of the catalysts is critical for achieving the desired electrocatalytic properties. Lu et al. [62] demonstrated the design of AuCo nanoparticles as catalysts for OER in alkaline media. The AuCo nanoparticles exhibit a uniform size distribution with a core–shell structure (Fig. 13a). The catalysts showed a composition dependence of the activity, displaying a maximum OER activity for Au:Co ratio of 2:3, as shown in Fig. 13c [62]. The AuCo nanoparticles are partially alloyed with segregated phases of fcc Au, hcp Co, and fcc Co, as detected by XRD. The surface partially phases segregated sites of the AuCo nanoparticles are shown to exhibit a bifunctional synergy of Co and Au where Co acts as active center in a higher valent state whereas the surface Au serves as a strong electron sink promoting various steps of OER (Fig. 13b).

#### Non-noble metal based electrocatalysts

Noble-metal-free OER electrocatalysts have attracted considerable research interest because of their low-cost and abundant supplies. Increasing efforts have been devoted to looking for efficient noble-metal-free OER electrocatalysts. Significant progress has been made in the past decades in demonstrating superb catalytic activity comparable to the noble metal catalyst. In this subsection, we will highlight some recent strategies in terms of increasing active sites by controlling the morphology, manipulating the composition, tuning the electronic structure and binding energy of the intermediates through elemental doping, defect engineering, and enhancing electroconductivity and electron transport through incorporating hybrid structures into composites for the rational design highly efficient OER electrocatalysts.

**Fig. 14:**
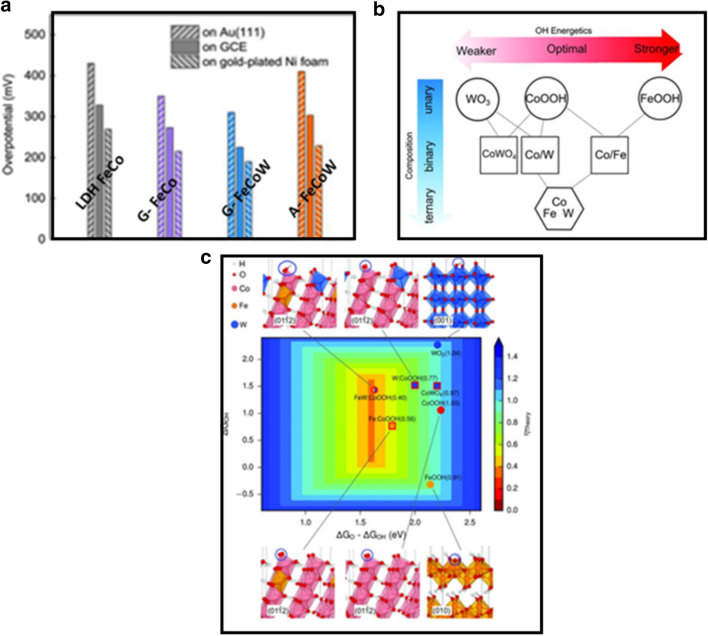
**a** Overpotentials obtained from OER polarization curves on Au(111), GCE (glass carbon electrode), and gold-plated Ni foam at 10 mA cm^−2^. **b** Illustration of tuning the energetics of OER intermediates on CoOOH via alloying with Fe and W. **c** Map of OER activities for pure Fe, Co oxyhydroxides and W, Fe-doped Co oxyhydroxides, cobalt tungstate, and W oxides calculated with DFT + U (Figure reprinted with permission from Ref. [63])

Recently, Zhang et al. [63] prepared a gelled FeCoW oxyhydroxide (W, Fe-doped CoOOH, G-FeCoW) with homogeneous metal distribution by sol–gel fabrication. The FeCoW oxyhydroxide exhibited the lowest overpotential of 191 mV at a current density of 10 mA cm^−2^ and 500 h cycle stability. This performance is superior to that of the benchmark Ni–Fe based catalyst. In Fig. 14a, the overpotentials at the current density of 10 mA cm^−2^ for Au(111), GCE (glass carbon electrode), and gold-plated Ni foam are compared. The result shows that the catalytic activity of G-FeCoW is much higher than that of the annealed A-FeCoW, gelled FeCo without W (G-FeCo), and LDH FeCo on different substrates. The synergistic effect of Fe and W co-doped Co oxyhydroxide allowed the optimal adsorption energy of intermediate OH, as supported by density function theory (DFT) plus Hubbard U correction, i.e., the DFT + U calculation, which is widely used in first-principles studies of some strongly-correlated systems (Fig. 14b). The calculated theoretical OER overpotential, as presented in the two-dimensional (2D) volcano plot (Fig. 14c), exhibits a significantly enhanced catalytic activity toward OER. A theoretical overpotential of only 0.4 V is derived by modulating the local electronic and geometrical environments.

**Fig. 15:**
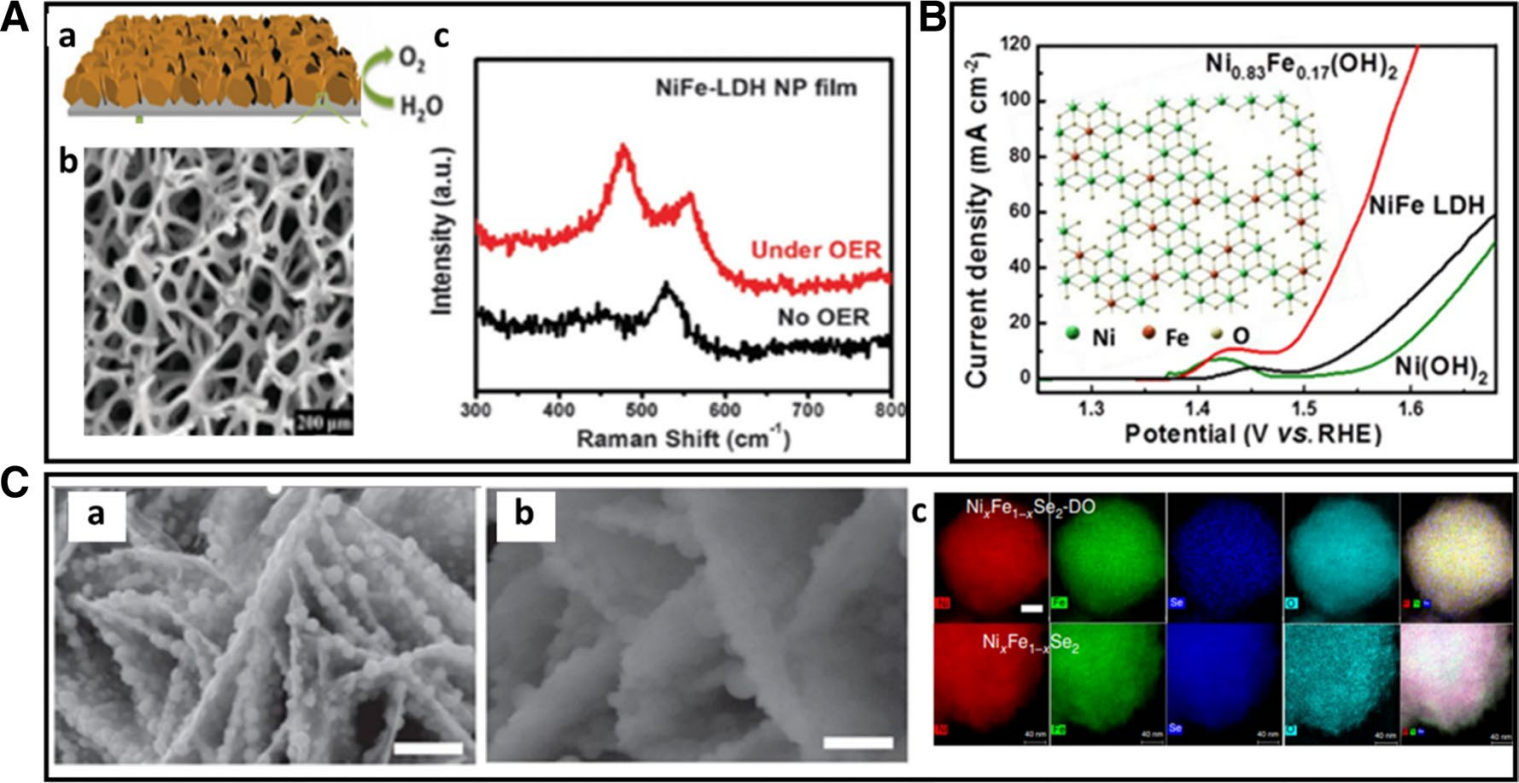
**A** (a) Schematic illustration of NiFe-LDH nanoplates grown on nickel foam, (b) SEM image of Ni foam, (c) in situ Raman spectra of NiFe-LDH films with and without OER operation (Figure reprinted with permission from Ref. [65]). **B** iR-corrected LSV polarization curves of Ni_0.83_Fe_0.17_(OH)_2_, NiFe LDH and Ni(OH)_2_ (Figure reprinted with permission from Ref. [66]). **C** SEM images of (a) Ni_x_Fe_1−x_Se_2_ and (b) Ni_x_Fe_1−x_Se_2_-Do (c) comparison of Ni_x_Fe_1−x_Se_2_ and Ni_x_Fe_1−x_Se_2_-Do in elemental mapping images (Figure reprinted with permission from Ref. [67])

Defect engineering is another effective pathway to regulate the structural and electronic properties of the electrocatalysts. The enhancement of the OER activity can be achieved by modulating the intermediate adsorption energy, which sometimes leads to unexpected active sites. Dai et al. reported a method to generate sufficient oxygen vacancies by plasma engraving strategy on Co_3_O_4_ nanosheet. SEM and TEM analysis of the catalysts revealed that the plasma engraved Co_3_O_4_ nanosheet has a rough, discontinuous, and loose surface with enhanced exposure of the surface area. Furthermore, Co^3+^ is partially reduced to Co^2+^ by Ar plasma treatment, producing oxygen vacancies, as confirmed by the X-ray photoelectron spectroscopy (XPS). This method not only can produce high surface area, but also modify its electronic structure by controlling Co^2+^/Co^3+^ ratio, leading to excellent OER catalytic activity with an overpotential of 153 eV at a current density of 10 mA cm^−2^ [64]. Besides the intrinsic alteration for the optimal absorption energy of intermediate species, the ability to tune the electron transport is crucial for improving the OER activity. Duan et al. performed in situ growth of 3D porous films of vertically aligned NiFe-LDH (layered double hydroxide) nanoplates (NiFe-LDH NPs) on nickel foams (Fig. 15a). The catalyst exhibits a small overpotential of 151 mV at 30 mA cm^−2^ better than 20 wt% Ir/C catalyst and prominent durability [65]. The observed high electrocatalytic activity was attributed to the synergistic effect of the 3D porous structure which provides a large surface area with a high density of active sites. The nickel foam substrate, as shown in Fig. 15A(b), is considered as an ideal substrate based on its porous structures and metallic electronic conductivity which accelerates the electron transport involved within OER. In situ Raman technique was employed to probe the active phase (Fig. 15A(c)). At an oxygen evolution potential, the detected new bands indicate the conversion of LDH into NiOOH, demonstrating NiOOH was the active phase for OER. The Fe incorporation into active site Ni hydroxide could create a more active site to enhance OER activity. Dou et al. [66] synthesized Fe-doped Ni(OH)_2_ nanosheet possessing a nanoporous surface structure with abundant defects by a facile and universal cation-exchange process which shows enhanced OER activity. In comparison with typical NiFe layered double hydroxide (LDH) nanosheets, the defect-rich Ni_0.83_Fe_0.17_(OH)_2_ nanosheets show the lowest overpotential of 245 mV at the current density of 10 mA cm^−2^. The excellent OER activity is attributed to a combination of the enriched active surface sites, abundant defects, and enhanced surface wettability. The success in using the cation-exchange process to prepare the active Fe-doped Co(OH)_2_ nanosheet has demonstrated a new pathway for the fabrication of highly effective OER catalysts.

The majority of non-precious metal catalysts include metal oxides and (oxy)hydroxides. Recently, many other promising electrocatalysts are exhibiting excellent OER catalytic activities, which consist of transition-metal phosphides, sulfides, and selenides. However, these compounds show unsatisfied stability under highly oxidative potential in alkaline solution. Therefore, the understanding of the chemical nature of the true active sites has attracted a great deal of attention in the development of OER-related catalysts. Hu and co-worker synthesized nanostructured nickel iron diselenide (Ni_x_Fe_1−x_Se_2_) as a templating precursor for in situ generations of a highly active nickel iron oxide catalyst. This catalyst showed excellent OER activity with an overpotential of only 195 mV for 10 mV cm^−2^ [67]. The SEM image analysis of Ni_x_Fe_1−x_Se_2_ as-synthesized and Ni_x_Fe_1-x_Se_2_ derived oxide (Ni_x_Fe_1−x_Se_2_-Do) after OER test, as shown in Fig. 15C(a, b), indicate that Ni_x_Fe_1−x_Se_2_-Do has an overall morphology similar to Ni_x_Fe_1−x_Se_2_ nanoparticles grown on the nanoplates. The elemental mapping of the change of composition after the in situ transformation from Ni_x_Fe_1−x_Se_2_ to Ni_x_Fe_1−x_Se_2_-Do (Fig. 15C(c)) showed that Se content was removed, the oxygen was incorporated in the structure, and Ni and Fe remained homogeneously distributed in the structure. Powder XRD and XPS analysis of the structure and composition of Ni_x_Fe_1−x_Se_2_ and Ni_x_Fe_1−x_Se_2_-Do confirmed the hypothesis that Ni_x_Fe_1−x_Se_2_ is partially transformed in situ into the corresponding metal hydroxides at the catalyst surface under OER conditions, which correspond to the real active OER sites.

### OER mechanism involving lattice oxygen species

In the conventional AEM, the entire reaction proceeds on a single metal site and there is a limitation by the scaling relation among the OER intermediates where the minimum theoretical overpotential is 0.37 eV. Recently, a new OER mechanism involving lattice oxygen species is proposed, i.e., lattice oxygen mediated mechanism (LOM) [68–71]. In LOM, the lattice oxygen on the catalyst directly participates in the oxygen evolution reaction. The participation of lattice oxygen is a phenomenon recently demonstrated for alloy catalysts in gas-phase catalytic oxidation reactions [72]. It is intriguing that this phenomenon is considered as an alternative reaction pathway, and sometimes the most favorable one, in the OER electrocatalysis.

**Fig. 16:**
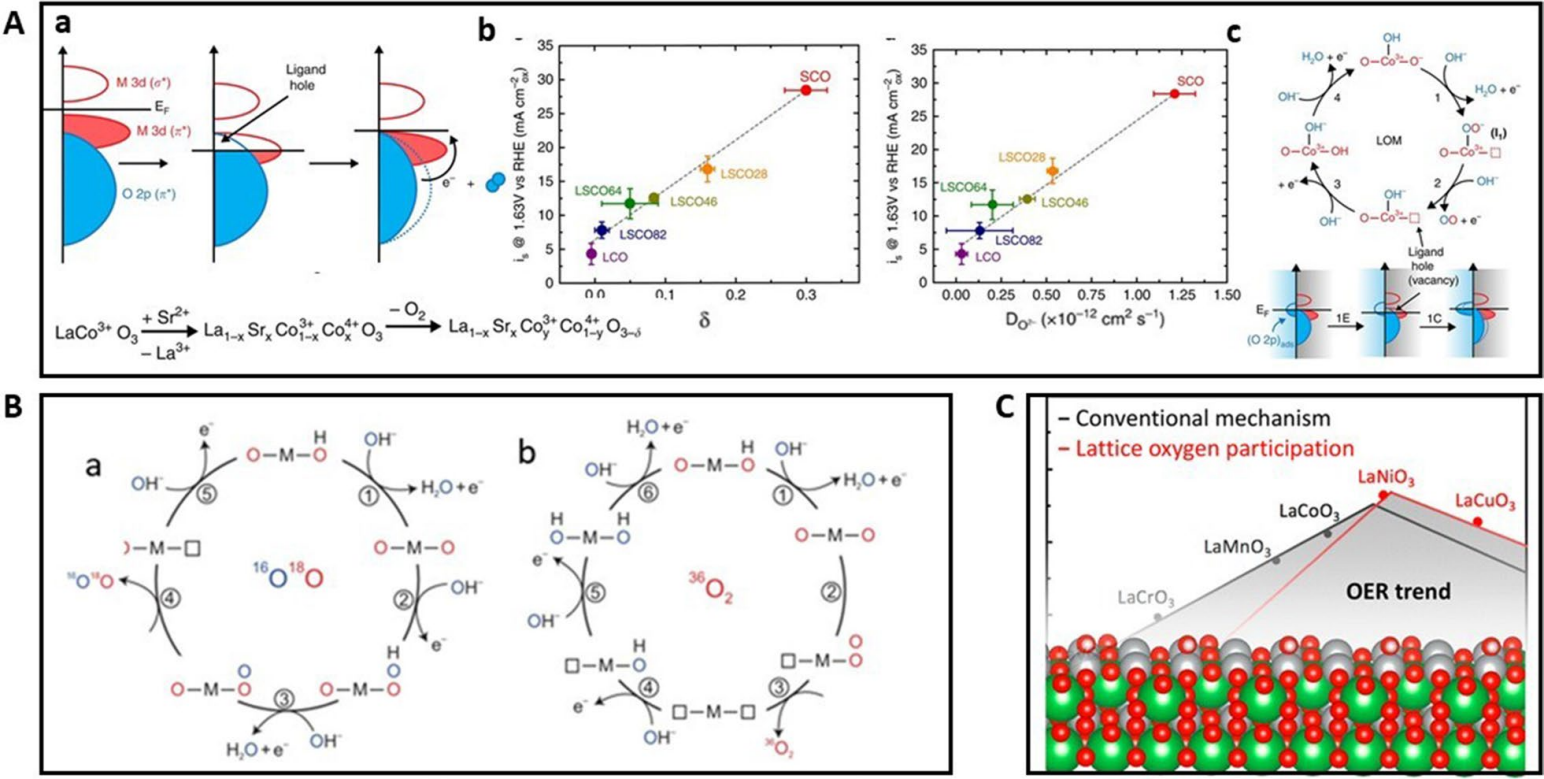
**A** (a) Illustration of oxygen vacancy concentration and Co–O bond covalency. (b) Relationship between oxygen vacancies, oxygen diffusivity, and the OER activity. (c) The proposed LOM mechanism (Figure reprinted with permission from Ref. [73]). **B** Possible OER mechanisms involving concerted proton-electron transfer on surface oxygen sites to yield (a) ^34^O_2_(^16^O^18^O), and (b) ^36^O_2_(^18^O^18^O), respectively (Figure reprinted with permission from Ref. [68]). **C** Volcano plot for OER on some perovskites with AEM and LOM (Figure reprinted with permission from Ref. [70])

Indeed, progress has been made by several key studies of the mechanism involving lattice oxygen species. Stevenson et al. [73] proposed a primary reaction pathway where the lattice oxygen participate in OER reaction via reversible formation of surface oxygen vacancies based on a DFT study. They presented a series of cobaltite perovskites structure and demonstrated the relationship between oxygen vacancies, metal–oxygen covalency, and OER activity. Figure 16A(a) shows the relationship between oxygen vacancy concentration and Co–O bond covalency. DFT studies have shown that by substituting Co^3+^ with Sr^2+^, the Fermi level (E_F_) moves closer to the O 2p band which is accompanied with an increase in the overlap between the M 3d band and O 2p band, indicating an enhanced covalency between metal–oxygen bond. At the same time, it creates ligand holes. The structural arrangement is followed to reduce the energy reaching a stable state by formation and release of O_2_, resulting in oxygen vacancies. Therefore, the enhanced covalency between metal–oxygen bond exhibits a higher vacancy concentration in the catalyst which can be controlled by substitution of the lower valence Sr^2+^ into La_1−*x*_Sr_*x*_CoO_3_−_δ_(LSCO) structure. The DFT modeling and experimental data results show a direct relationship between oxygen vacancies, oxygen diffusion rate, and the OER activity (Fig. 16A(b)). The data indicate that the increased vacancy and surface exchange kinetics correlate with the increased OER activity. Based on the correlation, a LOM mechanism is proposed as a parallel reaction mechanism to AEM, which is shown in Fig. 16A(c). The lattice oxygen reacts with the adsorbed oxygen on the metal site to form adsorbed –OO intermediates and leave an oxygen vacancy in the lattice. This is different from AEM mechanism involving the generally-proposed adsorbed –O species. For a given LSCO composition, the key to determining if the OER proceeds via the AEM or LOM is the relative stabilities of these two intermediates. It is predicted that there is a transition from the AEM to LOM upon increasing x in La_1−*x*_Sr_*x*_CoO_3−δ_ system which will reduce the O vacancy formation energy and reduce the bulk stability.

Using in situ ^18^O-isotope-labeling mass spectrometry, Shao-Horn et al. provide direct experimental evidence that the lattice oxygen is involved in the production of oxygen molecules during the OER. They demonstrated that in highly covalent metal oxides such as La_0.5_Sr_0.5_CoO_3_−_δ_ and SrCoO_3−δ_ (δ represents the vacancy parameter), the lattice oxygen enables to oxide formation during the OER process which involves non-concerted proton-electron transfer steps and exhibits pH-independent OER activity [68]. Therefore, the OER mechanism is triggered when metal–oxygen covalency is increased. On-line electrochemical mass spectrometry (OLEMS) is used to detect the participation of lattice oxygen in the oxidation reaction by ^18^O-labelled Co-based perovskites La_0.5_Sr_0.5_CoO_3−δ_, SrCoO_3−δ_, and Pr_0.5_Ba_0.5_CoO_3_−_δ_ which have different metal–oxygen covalency. Oxygen gas was measured in situ by mass spectroscopy in terms of different molecular weights such as ^32^O_2_(^16^O^16^O), ^34^O_2_(^16^O^18^O), and ^36^O_2_(^18^O^18^O). There are two different possible lattice oxygen species involved in the oxidation mechanism, which are proposed to explain the formation of ^34^O_2_(^16^O^18^O) and ^36^O_2_(^18^O^18^O), as shown in Fig. 16B. These steps all involved a chemical step to produce molecular O_2_ and the oxygen vacancy activity happening on the surface oxygenated sites. Furthermore, Kolpak et al. constructed AEM and LOM activity volcano plots as shown in Fig. 16C. The LOM exhibits a higher OER activity than AEM by minimizing the thermodynamically required overpotential [70]. For AEM, based on the scaling relation, there is a minimum theoretical overpotential of 0.37 eV. However, for LOM, the relative constant ΔG around 1.4 to 1.6 eV for *V*_O_ + OO* → *V*_O_ + OH* is much smaller than the AEM-based ΔG, which is 3.2 eV. Therefore, the minimum OER overpotential is only 0.17 eV for LOM, demonstrating a new avenue for the design of better OER electrocatalysts.

## Summary and perspectives

Taken together, significant progress has been made in the design of electrocatalysts for water splitting for the production of hydrogen by advancing the atomic, molecular, and nanoscale materials engineering strategies. Hydrogen is a promising substitute for fossil fuel as its highest gravimetric energy density and zero pollution emission, which provides a clean and renewable energy as an alternative to fossil fuels. The development of water splitting cells as an efficient energy conversion and storage system play an important role in hydrogen production. However, the energy efficiencies of water electrolysis are hindered by the sluggish reaction kinetics of OER and HER due to high overpotentials which lead to only 4% of the word’s hydrogen generation from water splitting at present. To facilitate the practical use of water splitting in industries, the design of efficient catalysts plays a major role in both OER and HER to minimize the overpotential and improve the energy efficiencies. In this review, we highlighted some of the significant advances in the development of nanostructured noble-metal-based and non-noble-metal-based electrocatalysts for HER and OER (see Tables 1, 2), which show high performance approaching benchmark catalysts Pt and IrO_2_/RuO_2_ for the HER and OER with low cost. Fundamental insights have been gained into the mechanistic details of the synergistic structure, morphology, and composition of the catalysts for HER and OER in different media. The current understanding of the reaction mechanisms for HER and OER, especially the emerging LOM for OER, could lead to new advances in overcoming the limitation of the traditional AEM by providing a new avenue for the design of better OER electrocatalysts.

Despite the significant progress in understanding the electrocatalytic processes of the OER and HER, several challenges remain for the ultimate commercial large-scale production of hydrogen by water splitting electrolysis. First, the development of non-noble metal OER electrocatalysts with high activity and long-term stability performance in acidic media remains a challenging area of research and development. This challenge stems from the increasing use of proton exchange membrane (PEM) electrolysis is promising water electrolysis because of PEM’s high energy efficiency and fast hydrogen production rate. PEM electrolysis for water splitting is operated under acidic conditions. For HER, there is various efficient non-noble metal electrocatalyst available in acid media. However, for OER, most of the efficient OER catalyst is Ir and Ru based electrocatalysts which have higher dissolution resistance in acidic condition. For non-noble metal-based electrocatalysts, most of them, cannot survive under such conditions. Thus, there is a clear need for the development of stable and robust non-noble metal OER electrocatalysts. Recently, Nocera et al. demonstrated a rational approach for designing non-noble metal based electrocatalysts that exhibit high activity and stability in acidic media by treating activity and stability as a decoupled elements of mixed metal oxides. For example, manganese is used as a stabilizing structural element which is coupled to the catalytically active Co center in CoMnO_x_ film [29]. Secondly, there is a limited knowledge of the detailed catalytic mechanisms especially for transition-metal-based HER and OER electrocatalysts. The intrinsic active site of electrocatalysts cannot be completely determined based on the descriptor of TOF. Recently, non-noble-metal-based carbides, phosphides, and chalcogenides have drawn great attention due to their high performance for OER in alkaline media. However, the nanostructured electrocatalysts undergo composition and structural transformations during the reaction under OER conditions. Therefore, understanding the structural transformation is required to determine the real active phases and sites. Gaining insight into the detailed mechanism, structural transformation, real active sites is critical for the rational design of the optimal performance catalysts. Integration of in situ characterization techniques and theoretical modeling is an advanced approach to gain insights into the structural transformation, reaction intermediates, and reaction pathways of the catalysts. Thirdly, it is difficult to directly compare various nanostructured catalyst materials based on the performance descriptors due to the different mass loadings of the catalysts on the electrode and the different materials of substrate which may affect the electron transfer rate by different electrochemical measurement methods. More effective electrocatalysts screening strategies are needed to establish the standard evaluation protocol for effective comparisons of the performances of catalysts from different research groups. Nevertheless, the surge of recent interests in nanostructure and lattice oxygen engineering of catalysts is expected to lead to new advances in the design of active, stable, and low-cost OER and HER electrocatalysts for the mass commercialization of water-splitting based hydrogen production.

## Data Availability

Not applicable.

## References

[1] Khan MA, Zhao H, Zou W, Chen Z, Cao W, Fang J, Xu J, Zhang L, Zhang J (2018). Electrochem. Energy Rev..

[2] Li A, Sun Y, Yao T, Han H (2018). Chem. Eur. J..

[3] Zhu J, Hu L, Zhao P, Lee LYS, Wong K-Y (2019). Chem. Rev..

[4] Song J, Wei C, Huang Z-F, Liu C, Zeng L, Wang X, Xu ZJ (2020). Chem. Soc. Rev..

[5] Suen N-T, Hung S-F, Quan Q, Zhang N, Xu Y-J, Chen HM (2017). Chem. Soc. Rev..

[6] Yu F, Yu L, Mishra I, Yu Y, Ren Z, Zhou H (2018). Mater. Today Phys..

[7] Zou X, Zhang Y (2015). Chem. Soc. Rev..

[8] Hu C, Zhang L, Gong J (2019). Energy Environ. Sci..

[9] Wu ZP, Lu XF, Zang SQ, Lou XW (2020). Adv. Funct. Mater..

[10] Fabbri E, Schmidt TJ (2018). ACS Catal..

[11] Yan Y, Xia BY, Zhao B, Wang X (2016). J. Mater. Chem. A.

[12] Zhong CJ, Luo J, Fang B, Wanjala BN, Njoki PN, Loukrakpam R, Yin J (2010). Nanotechnology.

[13] Loukrakpam R, Luo J, He T, Chen Y, Xu Z, Njoki PN, Wanjala BN, Fang B, Mott D, Yin J, Klar J, Powell B, Zhong CJ (2011). J. Phys. Chem. C.

[14] Wang W, Wang Z, Wang J, Zhong CJ, Liu CJ (2017). Adv. Sci..

[15] Zhong CJ, Luo J, Njoki PN, Mott D, Wanjala B, Loukrakpam R, Lim S, Wang L, Fang B, Xu Z (2008). Energy Environ. Sci..

[16] Jiang R, on Tung S, Tang Z, Li L, Ding L, Xi X, Liu Y, Zhang L, Zhang J (2018). Energy Storage Mater..

[17] Zhang C, Shen X, Pan Y, Peng Z (2017). Front. Energy Res..

[18] Sui S, Wang X, Zhou X, Su Y, Riffat S, Liu CJ (2017). J. Mater. Chem. A.

[19] Wu ZP, Caracciolo DT, Maswadeh Y, Wen J, Kong Z, Shan S, Vargas JA, Yan S, Hopkins E, Park K, Sharma A, Ren Y, Petkov V, Wang L, Zhong CJ (2021). Nat. Commun..

[20] Chang F, Bai Z, Li M, Ren M, Liu T, Yang L, Zhong CJ, Lu J (2020). Nano Lett..

[21] Kong Z, Maswadeh Y, Vargas JA, Shan S, Wu ZP, Kareem H, Leff AC, Tran DT, Chang F, Yan S, Nam S, Zhao XF, Lee JM, Luo J, Shastri S, Yu G, Petkov V, Zhong CJ (2019). J. Am. Chem. Soc..

[22] Wu ZP, Shan S, Xie Z-H, Kang N, Park K, Hopkins E, Yan S, Sharma A, Luo J, Wang J, Petkov V, Wang LC, Zhong CJ (2018). ACS Catal..

[23] Xie Y, Yang Y, Muller DA, Abruña HD, Dimitrov N, Fang J (2020). ACS Catal..

[24] Wu ZP, Shan S, Zang SQ, Zhong CJ (2020). Acc. Chem. Res..

[25] Suib SL (2013). New and Future Developments in Catalysis: Batteries, Hydrogen Storage and Fuel Cells.

[26] Shan S, Luo J, Wu J, Kang N, Zhao W, Cronk H, Zhao Y, Joseph P, Petkov V, Zhong CJ (2014). RSC Adv..

[27] Sardar K, Petrucco E, Hiley CI, Sharman JD, Wells PP, Russell AE, Kashtiban RJ, Sloan J, Walton RI (2014). Angew. Chem. Int. Ed..

[28] Strmcnik D, Uchimura M, Wang C, Subbaraman R, Danilovic N, Van Der Vliet D, Paulikas AP, Stamenkovic VR, Markovic NM (2013). Nat. Chem..

[29] Huynh M, Ozel T, Liu C, Lau EC, Nocera DG (2017). Chem. Sci..

[30] Skúlason E, Karlberg GS, Rossmeisl J, Bligaard T, Greeley J, Jónsson H, Nørskov JK (2007). PCCP.

[31] Markovića NM, Sarraf ST, Gasteiger HA, Ross PN (1996). J. Chem. Soc. Faraday Trans..

[32] Trasatti S (1972). J. Electroanal. Chem. Interfacial Electrochem..

[33] Cook TR, Dogutan DK, Reece SY, Surendranath Y, Teets TS, Nocera DG (2010). Chem. Rev..

[34] Sheng W, Myint M, Chen JG, Yan Y (2013). Energy Environ. Sci..

[35] Xie L, Liu Q, Shi X, Asiri AM, Luo Y, Sun X (2018). Inorg. Chem. Front..

[36] Zhao Z, Liu H, Gao W, Xue W, Liu Z, Huang J, Pan X, Huang Y (2018). J. Am. Chem. Soc..

[37] Xie Y, Cai J, Wu Y, Zang Y, Zheng X, Ye J, Cui P, Niu S, Liu Y, Zhu J (2019). Adv. Mater..

[38] Xiao P, Yan Y, Ge X, Liu Z, Wang J-Y, Wang X (2014). Appl. Catal. B.

[39] Gao W, Shi Y, Zhang Y, Zuo L, Lu H, Huang Y, Fan W, Liu T (2016). ACS Sustain. Chem. Eng..

[40] Popczun EJ, McKone JR, Read CG, Biacchi AJ, Wiltrout AM, Lewis NS, Schaak RE (2013). J. Am. Chem. Soc..

[41] Zhang R, Wang X, Yu S, Wen T, Zhu X, Yang F, Sun X, Wang X, Hu W (2017). Adv. Mater..

[42] Xie J, Zhang H, Li S, Wang R, Sun X, Zhou M, Zhou J, Lou XW, Xie Y (2013). Adv. Mater..

[43] Faber MS, Dziedzic R, Lukowski MA, Kaiser NS, Ding Q, Jin S (2014). J. Am. Chem. Soc..

[44] Xie J, Zhang J, Li S, Grote F, Zhang X, Zhang H, Wang R, Lei Y, Pan B, Xie Y (2013). J. Am. Chem. Soc..

[45] Wang Z, Ren X, Luo Y, Wang L, Cui G, Xie F, Wang H, Xie Y, Sun X (2018). Nanoscale.

[46] Li Q, Wu L, Wu G, Su D, Lv H, Zhang S, Zhu W, Casimir A, Zhu H, Mendoza-Garcia A (2015). Nano Lett..

[47] Huang X-Y, Wang AJ, Zhang L, Fang K-M, Wu L-J, Feng JJ (2018). J. Colloid Interface Sci..

[48] Subbaraman R, Tripkovic D, Strmcnik D, Chang K-C, Uchimura M, Paulikas AP, Stamenkovic V, Markovic NM (2011). Science.

[49] Levy R, Boudart M (1973). Science.

[50] Kitchin JR, Nørskov JK, Barteau MA, Chen JG (2005). Catal. Today..

[51] Vrubel H, Hu X (2012). Angew. Chem. Int. Ed..

[52] Liu P, Rodriguez JA (2005). J. Am. Chem. Soc..

[53] Hinnemann B, Moses PG, Bonde J, Jørgensen KP, Nielsen JH, Horch S, Chorkendorff I, Nørskov JK (2005). J. Am. Chem. Soc..

[54] Jaramillo TF, Jørgensen KP, Bonde J, Nielsen JH, Horch S, Chorkendorff I (2007). Science.

[55] Nørskov JK, Rossmeisl J, Logadottir A, Lindqvist L, Kitchin JR, Bligaard T, Jonsson H (2004). J. Phys. Chem. B.

[56] Man IC, Su HY, Calle-Vallejo F, Hansen HA, Martinez JI, Inoglu NG, Kitchin J, Jaramillo TF, Nørskov JK, Rossmeisl J (2011). ChemCatChem.

[57] Nørskov JK, Abild-Pedersen F, Studt F, Bligaard T (2011). PNAS.

[58] Su J, Ge R, Jiang K, Dong Y, Hao F, Tian Z, Chen G, Chen L (2018). Adv. Mater..

[59] Wang Y, Zhang L, Yin K, Zhang J, Gao H, Liu N, Peng Z, Zhang Z (2019). ACS Appl. Mater. Interfaces.

[60] Park J, Sa YJ, Baik H, Kwon T, Joo SH, Lee K (2017). ACS Nano.

[61] Lim J, Park D, Jeon SS, Roh CW, Choi J, Yoon D, Park M, Jung H, Lee H (2018). Adv. Funct. Mater..

[62] Lu A, Peng D-L, Chang F, Skeete Z, Shan S, Sharma A, Luo J, Zhong CJ (2016). ACS Appl. Mater. Interfaces.

[63] Zhang B, Zheng X, Voznyy O, Comin R, Bajdich M, García-Melchor M, Han L, Xu J, Liu M, Zheng L (2016). Science.

[64] Xu L, Jiang Q, Xiao Z, Li X, Huo J, Wang S, Dai L (2016). Angew. Chem. Int. Ed..

[65] Lu Z, Xu W, Zhu W, Yang Q, Lei X, Liu J, Li Y, Sun X, Duan X (2014). Chem. Commun..

[66] Zhou Q, Chen Y, Zhao G, Lin Y, Yu Z, Xu X, Wang X, Liu HK, Sun W, Dou SX (2018). ACS Catal..

[67] Xu X, Song F, Hu X (2016). Nat. Chem..

[68] Grimaud A, Diaz-Morales O, Han B, Hong WT, Lee Y-L, Giordano L, Stoerzinger KA, Koper MT, Shao-Horn Y (2017). Nat. Chem..

[69] Zhu Y, Tahini HA, Hu Z, Chen ZG, Zhou W, Komarek AC, Lin Q, Lin HJ, Chen CT, Zhong Y (2020). Adv. Mater..

[70] Yoo JS, Rong X, Liu Y, Kolpak AM (2018). ACS Catal..

[71] Huang ZF, Song J, Du Y, Xi S, Dou S, Nsanzimana JMV, Wang C, Xu ZJ, Wang X (2019). Nat. Energy.

[72] Shan S, Li J, Maswadeh Y, O’Brien C, Kareem H, Tran DT, Lee IC, Wu ZP, Wang S, Yan S, Cronk H, Mott D, Yang L, Luo J, Petkov V, Zhong CJ (2020). Nat. Commun..

[73] Mefford JT, Rong X, Abakumov AM, Hardin WG, Dai S, Kolpak AM, Johnston KP, Stevenson KJ (2016). Nat. Commun..

